# Measuring Environmental and Behavioral Drivers of Chronic Diseases Using Smartphone-Based Digital Phenotyping: Intensive Longitudinal Observational mHealth Substudy Embedded in 2 Prospective Cohorts of Adults

**DOI:** 10.2196/55170

**Published:** 2024-10-11

**Authors:** Li Yi, Jaime E Hart, Marcin Straczkiewicz, Marta Karas, Grete E Wilt, Cindy R Hu, Rachel Librett, Francine Laden, Jorge E Chavarro, Jukka-Pekka Onnela, Peter James

**Affiliations:** 1 Department of Nutrition, Harvard T.H. Chan School of Public Health Boston, MA United States; 2 Department of Population Medicine Harvard Medical School and Harvard Pilgrim Health Care Institute Boston, MA United States; 3 Department of Environmental Health, Harvard T.H. Chan School of Public Health Boston, MA United States; 4 Channing Division of Network Medicine, Department of Medicine, Brigham and Women's Hospital and Harvard Medical School Boston, MA United States; 5 Department of Biostatistics, Harvard T.H. Chan School of Public Health Boston, MA United States; 6 Department of Public Health Sciences, School of Medicine, University of California Davis Davis, CA United States

**Keywords:** big data, daily mobility, digital phenotyping, ecological momentary assessment, epidemiological monitoring, health behavior, smartphone apps and sensors, mobile phone

## Abstract

**Background:**

Previous studies investigating environmental and behavioral drivers of chronic disease have often had limited temporal and spatial data coverage. Smartphone-based digital phenotyping mitigates the limitations of these studies by using intensive data collection schemes that take advantage of the widespread use of smartphones while allowing for less burdensome data collection and longer follow-up periods. In addition, smartphone apps can be programmed to conduct daily or intraday surveys on health behaviors and psychological well-being.

**Objective:**

The aim of this study was to investigate the feasibility and scalability of embedding smartphone-based digital phenotyping in large epidemiological cohorts by examining participant adherence to a smartphone-based data collection protocol in 2 ongoing nationwide prospective cohort studies.

**Methods:**

Participants (N=2394) of the Beiwe Substudy of the Nurses’ Health Study 3 and Growing Up Today Study were followed over 1 year. During this time, they completed questionnaires every 10 days delivered via the Beiwe smartphone app covering topics such as emotions, stress and enjoyment, physical activity, access to green spaces, pets, diet (vegetables, meats, beverages, nuts and dairy, and fruits), sleep, and sitting. These questionnaires aimed to measure participants’ key health behaviors to combine them with objectively assessed high-resolution GPS and accelerometer data provided by participants during the same period.

**Results:**

Between July 2021 and June 2023, we received 11.1 TB of GPS and accelerometer data from 2394 participants and 23,682 survey responses. The average follow-up time for each participant was 214 (SD 148) days. During this period, participants provided an average of 14.8 (SD 5.9) valid hours of GPS data and 13.2 (SD 4.8) valid hours of accelerometer data. Using a 10-hour cutoff, we found that 51.46% (1232/2394) and 53.23% (1274/2394) of participants had >50% of valid data collection days for GPS and accelerometer data, respectively. In addition, each participant submitted an average of 10 (SD 11) surveys during the same period, with a mean response rate of 36% across all surveys (SD 17%; median 41%). After initial processing of GPS and accelerometer data, we also found that participants spent an average of 14.6 (SD 7.5) hours per day at home and 1.6 (SD 1.6) hours per day on trips. We also recorded an average of 1046 (SD 1029) steps per day.

**Conclusions:**

In this study, smartphone-based digital phenotyping was used to collect intensive longitudinal data on lifestyle and behavioral factors in 2 well-established prospective cohorts. Our assessment of adherence to smartphone-based data collection protocols over 1 year suggests that adherence in our study was either higher or similar to most previous studies with shorter follow-up periods and smaller sample sizes. Our efforts resulted in a large dataset on health behaviors that can be linked to spatial datasets to examine environmental and behavioral drivers of chronic disease.

## Introduction

### Background

Physical activity, diet, and obesity are important behavioral risk factors for numerous noncommunicable diseases, such as coronary heart disease, type 2 diabetes, and certain cancers [1-5]. However, >80% of American adults do not adhere to weekly recommendations for aerobic and muscle-strengthening exercise [6], and their dietary habits remain suboptimal according to Dietary Guidelines for Americans standards [7]. Health behaviors are influenced by various environmental factors, including the built environment, food accessibility, and green spaces [8,9]. Well-designed neighborhoods provide infrastructure such as parks and sidewalks that enable regular physical activity [10,11], and green spaces mitigate exposure to noise, light pollution, and air pollution, thereby fostering a health-promoting environment [12-16]. Moreover, access to healthy food may promote better eating habits [17]. Research examining the effects of environmental factors on health behaviors is often limited by cross-sectional study designs and coarse data resolution. Such studies typically rely on participants’ residential addresses, neglecting time spent in other locations [18,19], and self-report measures of health behaviors, which lack objective information on the timing or duration of, for instance, physical activity and food intake [20]. These factors contribute to potential biases in assessing the influence of the environment on health behaviors. More recently, studies have begun to use mobile health (mHealth) technologies such as wearables and smartphone apps to capture location and behavioral data at a much higher spatial and temporal resolution (eg, minute-level location and second-level movement) [18,21-24]. However, these studies tend to be of short duration and have small convenience samples, which limits their representativeness [25,26]. For example, participants might have different levels of physical activity in the 2-week sample periods than in their usual routine. As another example, daily mobility behavior recorded over 7 days could omit less frequent routine behaviors such as grocery shopping.

Emerging data collection technologies such as smartphone-based digital phenotyping offer the opportunity to mitigate the limitations of the aforementioned studies using intensive data collection schemes by capitalizing on the widespread use of smartphones [27,28]. Smartphone-based digital phenotyping refers to the technology that quantifies human phenotypes at the individual level on a moment-to-moment basis using data from personal digital devices, particularly smartphones, to assess behavioral patterns, social interactions, and physical mobility [27]. Compared with studies that rely on mailing wearables, the smartphone-based approach to digital phenotyping allows for less burdensome data collection (eg, secure uploading of near–real-time passive GPS and accelerometer data) and lower costs. Consequently, digital phenotyping studies can achieve longer follow-up periods (eg, >1 year) compared to typical data collection periods (7-14 days) for intensive longitudinal studies [11,26,29]. Smartphone GPS data with high accuracy (eg, <50 m) can be merged with spatial datasets containing information on built and natural surroundings, noise levels, and air pollution to produce customized exposure metrics for environmental factors that change throughout the day over weeks and even months [29-31]. In addition, although the smartphone accelerometer may provide less complete data compared to commercial-grade accelerometry (eg, ActiGraph) because it usually uses noncontinuous data sampling schemes to conserve the smartphone battery, it is still capable of taking precise measurements (eg, 10 measures per second) that can provide an objective indicator of physical activity by identifying steps and cadence using advanced walk detection algorithms [32,33]. Smartphone apps can also be programmed to administer surveys on health behaviors or psychological health outcomes [14,34]. More importantly, these datasets can be linked at different temporal scales (eg, minute by minute and daily) and sequences (eg, instantaneous and time lapsed) to examine the relationships between environmental and behavioral factors and chronic disease outcomes.

### Objectives

In this paper, we present our assessment of the feasibility and scalability of embedding smartphone-based digital phenotyping in large epidemiological cohorts by examining adherence to smartphone-based digital phenotyping data collection over 1 year in 2 ongoing nationwide prospective cohort studies: the Nurses’ Health Study 3 (NHS3) and the Growing Up Today Study (GUTS). We also present our methodology for applying a smartphone-based digital phenotyping platform to continuously collect, manage, and process a substantial amount of smartphone-based GPS, accelerometer, and survey data involving approximately 2400 participants. Finally, we present selected mobility and physical activity outcomes derived from the processed high-spatial and temporal resolution GPS and accelerometer data, as well as results from diet, lifestyle (eg, visits to green spaces), and psychological well-being surveys, and discuss their potential applications for novel epidemiological investigations through different linkage schemes between different types of smartphone data and further link to the health behaviors and data collected from the ongoing NHS3 or GUTS prospective cohorts.

## Methods

### Beiwe Smartphone Substudy of the NHS3 and GUTS

The NHS3 is an ongoing web-based open cohort study of male and female nurses and nursing students in the United States and Canada that began in 2010, whereas the GUTS (N=27,706) is a closed cohort of children of participants in the Nurses’ Health Study II from the United States, which started in 1996 and expanded in 2004 [35]. To be eligible for the NHS3, participants must be registered nurses, licensed practical or vocational nurses, or nursing students and be born on or after January 1, 1965. For the GUTS, the participants were children of Nurses’ Health Study II participants. As of July 2021, a total of 35,567 participants in the NHS3 and 10,554 participants in the GUTS remained active. NHS3 participants complete questionnaires about their health and wellness every 6 months, whereas GUTS participants complete questionnaires every 1 to 3 years [35].

The Beiwe Smartphone Substudy (hereafter referred to as the substudy) involved a subset of NHS3 and GUTS participants (1703/2394, 71.14% from the NHS3 and 691/2394, 28.86% from the GUTS) who downloaded and registered on a smartphone app called Beiwe [36]. This substudy aimed to quantify the health impacts of environmental exposures, physical activity, and sleep using smartphone-collected GPS and accelerometer data and microsurveys. It served as a pilot to expand web-based mHealth technology and big data capabilities to the full NHS3 and GUTS cohorts. To be eligible for the substudy, participants recruited from the NHS3 must have completed at least 2 main study questionnaires and expressed interest in a biospecimen collection. GUTS participants must be current responders and have answered “yes” to any of the 3 biospecimen collection questions on the 2019 questionnaire. For the eligible participants in both cohorts to be invited, they must have an active email address on file and have not been actively enrolled in another NHS3 or GUTS substudy.

Invited participants were then asked to complete a screener for additional eligibility criteria. These criteria included (1) owning a smartphone, (2) having weekly Wi-Fi access, and (3) living in the 48 contiguous states of the United States. After that, invited participants who met the criteria were sent an electronic consent form. After providing their consent, they were instructed to download the Beiwe app on their smartphones. Multimedia Appendix 1 illustrates the sequences of different recruitment stages. The app enables the distribution of short surveys and high-resolution smartphone accelerometer and GPS data. In this substudy, participants were asked to participate in a sampling period of a year. Unlike the typical 7-day measurement lengths in other physical activity and GPS studies [29], this yearly protocol aimed to record minute-by-minute behaviors and exposures on workdays and nonworkdays and across weeks, months, and seasons. During the sampling period, participants were asked to keep their smartphones with them during waking hours and at home at night, synchronizing their data using Wi-Fi at least once a week. Enrollment for the substudy began in July 2021 and ended in August 2022. Data collection ended on June 15, 2023, defined as the last day for any study participants to transmit any data.

### Data Collection

Once downloaded, the Beiwe app guides users through the onboarding process and obtains consent to access location service data and send notifications. Multimedia Appendix 2 shows screenshots of the app page intended for participants. At the beginning of the sampling period, participants completed a short smartphone-based survey about pet ownership. Smartphone-based surveys were delivered every 10 days starting in July 2021 until the end of the sampling period for the last substudy participant. The topics of the surveys changed each time on a predetermined schedule and included surveys on sitting, physical activity, sleep, mood, stress and enjoyment, individual sections from food frequency questionnaires (eg, vegetables, meat, beverages, nuts and dairy, and fruits), and green space visits. The list of questions asked in each survey is shown in Multimedia Appendix 3.

Simultaneously, the Beiwe app uses the smartphone GPS data to estimate the location of the participants at 15-minute intervals (90 seconds on and 810 seconds off) [37]. In addition, the app uses the participants’ smartphone accelerometer to measure triaxial acceleration at a preconfigured frequency of 10 Hz (ie, 90-second intervals with 30 seconds on and 60 seconds off) [25]. Interval-based collection of GPS and accelerometer data was chosen to minimize battery consumption while maintaining the ability to determine participant movement patterns and behavior throughout the day.

### Data Processing

The Beiwe app generates a large amount of data, including participants’ survey responses and GPS and accelerometer data, which were stored and processed on Amazon Web Services (AWS) servers. Considering the disparities in data characteristics (eg, passive sensing data vs survey questionnaires) and volumes (raw accelerometer data are much larger than GPS data), we developed distinct workflows to process the 3 main data streams of interest: GPS, accelerometer, and survey data.

#### GPS Data

The raw GPS data gathered at a fixed sampling rate of approximately every 15 minutes underwent Python-based preprocessing and imputation using a statistical method developed specifically for Beiwe data named Jasmine in the *Forest* Python library (Python version 3.9.2; Python Software Foundation) [37]. This algorithm first filtered out participants with insufficient data and location coordinates with <50 meters of horizontal accuracy based on previous literature examining the quality of GPS data collected via the Beiwe app [37]. The algorithm first identified flights (ie, periods of straight-line movement) and pauses (ie, periods of nonmovement) for each participant from the collected data. The algorithm then imputed missing data (ie, missing flights and pauses) based on resampling of observed flights and pauses using a bidirectional imputation approach based on a sparse Online Gaussian process. Finally, the algorithm converted the flights and pauses table into a record of minute-by-minute GPS coordinates using the start and end coordinates of the flights and pauses along with their durations. In addition to generating minute-level datasets, the algorithm calculated daily summary statistics that measured participants’ mobility, such as the time spent at home and on trips and number of places visited. The home location was inferred automatically from the data. A trip was defined as GPS trajectories between 2 locations at least 50 meters apart where participants stayed for at least 15 minutes. Places visited were defined as GPS point clusters that were at least 15 minutes in duration and 50 meters apart. The GPS imputation method has been implemented as open-source software and can be accessed through a GitHub link [38].

#### Accelerometer Data

For accelerometer data, first, raw accelerometer data collected by the Beiwe app were retrieved from the AWS production server and stored in the temporary storage of the AWS computing instance on the analytical server that we set up. The data were organized by participant and contained a list of files storing subsecond-level measurements of acceleration on the x-, y-, and z-axes at each hour. Second, these data were fed into a Python-based walking recognition algorithm named Oak in the *Forest* Python library that estimated gait cadence for each second of observation (ie, how many steps a person took in that second) [38]. This method, validated against 20 publicly available annotated datasets on walking activity data collected at various body locations (thigh, waist, chest, arm, and wrist), accurately estimates walking periods with high sensitivity and specificity [32]. Details of the validated walking detection algorithm for Beiwe can be found through a GitHub link [32]. Briefly, we aggregated second-level walking data to minutes, enabling alignment with the GPS data collected simultaneously.

#### Survey Data

The submitted raw survey responses were stored in 1 master file. To process the survey data, we first divided the file into 12 datasets, each corresponding to 1 of 12 surveys administered. The survey topics were sitting, stress and enjoyment, emotions, green space visits, physical activity, pets, sleep, vegetables, nuts and dairy, meats, beverages, and fruits. The datasets were further preprocessed to calculate metadata (eg, the time from survey start to survey submission) into table columns, duplicate survey submissions were eliminated, and empty survey submissions were removed. In addition, we summarized and visualized the response distributions for each survey question.

### Statistical Analysis Plan

In this manuscript, we present response rates for each step of substudy participant recruitment and show demographic characteristics (means and frequencies) compared to those of the full NHS3 and GUTS cohorts. Although data collection was ongoing, we also present preliminary statistics on compliance with the GPS, accelerometer, and survey data collection at both participant and participant day levels.

#### Participant Day–Level Compliance

Following the recommendation of a previous study that examined accelerometer data collected by the Beiwe app [32], we defined a valid second as one that had at least 9 observations and a valid minute as one that had at least one valid second. Similarly, for GPS data, we defined a 15-minute data collection segment as valid if at least one observation with <50 meters of horizontal accuracy was recorded. A valid day was defined as one with at least 600 valid minutes (10 valid hours) for GPS and accelerometer data, which is commonly used in physical activity studies using GPS and accelerometer devices [29]. The participant day–level compliance was calculated as the percentage of valid days out of the total potential data collection days. Potential data collection days for each participant were defined as the number of days between the date the participant registered on the app and the data download date selected for this study (if they had not yet completed a year of sampling) or the end date of the participant (if they had completed a year of sampling). For sensitivity analysis, we also calculated the participant day–level compliance using each participant’s follow-up time instead, which excluded the days after the participant deleted the Beiwe app. In addition, we calculated noncollection prevalence for both GPS and accelerometer data by calculating the percentage of days without data out of the total potential days. We also performed sensitivity analyses for the aforementioned statistics using more stringent criteria, which defined a valid day as one that had at least 1200 valid minutes (20 valid hours), which may be a better indicator of how well participants provided data for a full 24-hour period.

#### Participant-Level Compliance

At the participant level, GPS and accelerometer data collection compliance was defined as follows: (1) “excellent” if the participants had at least 75% of their potential data collection days as valid days, (2) “good” if they had between 50% and 75% of their potential data collection days as valid days, (3) “fair” if they had between 25% and 50% of their potential data collection days as valid days, and (4) “poor” if they had <25% of their potential data collection days as valid days. Regarding survey data, we computed the response rate for each survey sent over a year, which was equivalent to the total number of participants who completed and submitted the survey over the total number of potential surveys that a participant would have received. Finally, we also presented some preliminary statistics (means, medians, and IQRs) of some mobility and walking behavior metrics derived from the processed datasets. For mobility, we estimated daily time spent at home and on trips, total distance traveled, and total number of places visited derived from the GPS data; for walking behavior, we calculated daily steps (sum of steps at the minute level) and walking minutes (sum of total seconds within each minute in which we identified steps) derived from the accelerometer data; and, for the surveys, we summarized the answers to a selective list of questions asking about various environmental and behavioral drivers of chronic diseases.

In this substudy, participants were drawn from 2 large nationwide cohorts with potentially disparate demographic characteristics. In addition, evidence has indicated that smartphone apps installed on Android and iOS systems may function differently during the collection of GPS, accelerometer, and survey data because of the heterogeneity of the smartphone hardware and software [34]. Thus, to explore whether the cohort or smartphone type affected the various compliance criteria that we were calculating, we also broke down the aforementioned statistics by cohort and phone operating system (OS). All statistical analyses were performed in AWS using Python and the Forest library for raw Beiwe data processing and in R (version 4.1.0; R Foundation for Statistical Computing) for participant day– and participant-level data summaries and compliance statistics.

### Ethical Considerations

All procedures performed in studies involving human participants were in accordance with the ethical standards of the institutional review boards of the Brigham and Women’s Hospital and the Harvard TH Chan School of Public Health (protocol numbers 1999P002104 and 2006P000473). All participants provided informed consent for data collection at the beginning of the substudy. Participants were not compensated. Raw data collected by the Beiwe app were encrypted before being sent to the AWS production server. Subsequently, the data were either securely transferred to the analytical server (for GPS and survey data) or deleted from the temporary storage of the analytical server after processing (for accelerometer data). Both AWS production and analytics servers were protected by 2-factor authentication and SSP keys. To protect participant identity, no personal or health-related information was entered through Beiwe.

## Results

### Participant and Data Characteristics

The invitations to participate in the substudy were sent out starting on July 21, 2021. In total, we invited 32,441 NHS3 and GUTS participants to complete the eligibility screening. Of those 32,441 participants invited, 3470 (10.7%) completed the screener, and 3410 (98.27%) of these individuals were determined to be eligible (Multimedia Appendix 1). The comparison of demographics of participants who consented to the study versus those who did not, as well as participants whom we invited to complete eligibility screening versus those we did not invite, are shown in Multimedia Appendix 4. Overall, there was little difference across these groups. Of the 3410 eligible individuals, 2796 (81.99%) completed the consent process and received instructions to download the Beiwe app [36], and 2394 (70.21%) downloaded and registered on the app to participate in the substudy.

The demographics of the substudy compared with those of the entire NHS3 and GUTS cohorts are shown in Table 1. Overall, the sample had a mean age of 41.8 (SD 8.1) years and was predominantly female (2247/2394, 93.86%) and White (2243/2394, 93.69%). Over half (1437/2394, 60.03%) of the participants were married, and more than three-quarters (1817/2394, 75.9%) were nonsmokers. The mean BMI was 27.4 (SD 6.7) kg/m^2^. When comparing the characteristics of the substudy sample to those of the active NHS3 and GUTS cohorts at the beginning of the substudy, there were no major differences. More specifically, compared with the active NHS3 cohort, the substudy participants were older, more likely to be White individuals, and more likely to be nonsmokers and had a higher BMI but a similar distribution of sex (Table 1). In contrast, the substudy sample recruited from the GUTS were more likely to be female and less likely to be married and had a higher BMI than the active GUTS cohort. However, they had similar distributions for age, race, ethnicity, and smoking status (Table 1).

At the conclusion of our data collection period (June 2023), we had received 11.1 TB of data from 2394 participants, including 10.5 TB of accelerometer data, 243.5 GB of GPS data, and 23,682 survey submissions. By leveraging our cloud-based processing approach and customized Python-based algorithms (described in the Methods section), we were able to process our data, including GPS data processing and imputation, walking recognition, and cleaning of survey data. Our efforts eliminated the need to store a large amount of raw data locally and resulted in a much smaller processed dataset (eg, 1.9 GB of processed accelerometry data vs 10.5 TB of raw data), which substantially reduced data storage and processing time and costs.

**Table 1:** Demographic characteristics of active Nurses’ Health Study 3 (NHS3) and Growing Up Today Study (GUTS) cohorts at the beginning of the substudy (July 2021) and of participants enrolled in the Beiwe Smartphone Substudy.

Variable			Active GUTS cohort at the beginning of the substudy (n=10,554)		Active NHS3 cohort at the beginning of the substudy (n=35,567)		Beiwe Smartphone Substudy				
							All substudy participants (n=2394)		Participants from the GUTS (n=691)		Participants from the NHS3 (n=1703)
Age (y), mean (SD)			34.3 (3.5)		42.6 (7.8)		41.8 (8.1)		34.5 (3.5)		44.8 (7.5)
**Sex, n (%)**											
	Male	3453 (32.72)		474 (1.33)		147 (6.14)		115 (16.64)		32 (1.88)	
	Female	7101 (67.28)		35,093 (98.67)		2247 (93.86)		576 (83.36)		1671 (98.12)	
**Race, n (%)**											
	American Indian or Alaska native	43 (0.41)		456 (1.28)		22 (0.92)		5 (0.72)		17 (1)	
	Asian	214 (2.03)		1195 (3.36)		45 (1.88)		8 (1.16)		37 (2.17)	
	Black or African American	71 (0.67)		1159 (3.26)		51 (2.13)		6 (0.87)		45 (2.64)	
	Native Hawaiian or other Pacific Islander	40 (0.38)		148 (0.42)		7 (0.29)		2 (0.29)		5 (0.29)	
	White	10043 (95.16)		32002 (89.98)		2243 (93.69)		663 (95.95)		1580 (92.78)	
**Ethnicity, n (%)**											
	Hispanic or Latinx	249 (2.36)		1602 (4.5)		80 (3.34)		20 (2.89)		60 (3.52)	
	Not Hispanic or Latinx	10249 (97.11)		33804 (95.04)		2307 (96.37)		669 (96.82)		1638 (96.18)	
Married, n (%)			6594 (62.48)		20,646 (58.05)		1437 (60.03)		392 (56.73)		1045 (61.36)
**Smoking status, n (%)**											
	Never	7932 (75.16)		26,682 (75.02)		1817 (75.9)		528 (76.41)		1289 (75.69)	
	Current	346 (3.28)		1792 (5.04)		103 (4.3)		29 (4.2)		74 (4.35)	
	Former	2276 (21.56)		6809 (19.14)		468 (19.55)		134 (19.39)		334 (19.61)	
BMI (kg/m²), mean (SD)			26.7 (6)		26.7 (6.4)		27.4 (6.7)		27.2 (6.7)		27.5 (6.7)
**Phone OS** ^a^ **, n (%)**											
	Android	—^b^		—		645 (26.94)		217 (31.4)		428 (25.13)	
	iOS	—		—		1743 (72.81)		472 (68.31)		1271 (74.63)	
	Both^c^	—		—		6 (0.25)		2 (0.29)		4 (0.23)	

^a^OS: operating system.

^b^Not applicable.

^c^Some participants switched smartphones during the year-long data collection period, which resulted in a different OS.

### Participant and Data Collection Compliance

#### Overview

Overall, participants provided digital phenotyping data (at least one observation in a day) for an average of 53% (SD 39%; median 58%) of the total potential data collection days (ie, 1 year). On average, participants were followed up for 213 (SD 148; median 246) days, defined as the difference in days between the date of app registration and either the date when the participant deleted the app or the data download date, whichever came first. When breaking down by phone OS, the average follow-up time was slightly longer for iOS than Android users (223 days, SD 146 vs 187days, SD 149). A total of 77.99% (1867/2394) of the participants had at least 1 month of follow-up, 69.72% (1669/2394) of the participants had at least 3 months of follow-up, and 57.27% (1371/2394) had at least 6 months of follow-up. Table 2 provides a full list of metrics for the participant follow-up days. To explore the potential bias of our data due to participant attrition, we also compared the demographic characteristics of participants who had at least 1, 3, and 6 months of follow-up (Multimedia Appendix 5). The results indicated no major differences across groups.

At the participant day level, we collected an average of 14.8 (SD 5.9; median 14.3) valid hours of GPS data per day and 13.2 (SD 4.8; median 13.2) valid hours of accelerometer data per day. Android users had more valid hours of data per day than iOS users for both data types. Multimedia Appendix 6 provides details of smartphone device compliance.

**Table 2:** Percentages of participants who had follow-up lengthsa that were at least 7 days, 2 weeks, 1 month, 3 months, and 6 months (N=2394).

	Overall, n (%)	By phone OS^b^, n (%)		
		Android (n=645)	iOS (n=1743)	Both^c^ (n=6)
At least 7 days	2039 (85.17)	523 (81.09)	1510 (86.63)	6 (100)
At least 2 weeks	1954 (81.62)	501 (77.67)	1447 (83.02)	6 (100)
At least 1 month	1867 (78)	472 (73.18)	1390 (79.75)	5 (83.33)
At least 3 months	1669 (69.72)	405 (62.79)	1259 (72.23)	5 (83.33)
At least half a year	1371 (57.27)	324 (50.23)	1042 (59.78)	5 (83.33)

^a^Follow-up length was defined as the difference in days between the app registration date and either the last date on which the participant provided any data via the Beiwe app or the data download date of this study—November 1, 2022—whichever came first.

^b^OS: operating system.

^c^Some participants switched smartphones during the year-long data collection period, resulting in a different OS.

#### GPS and Accelerometer Data

**Figure 1:**
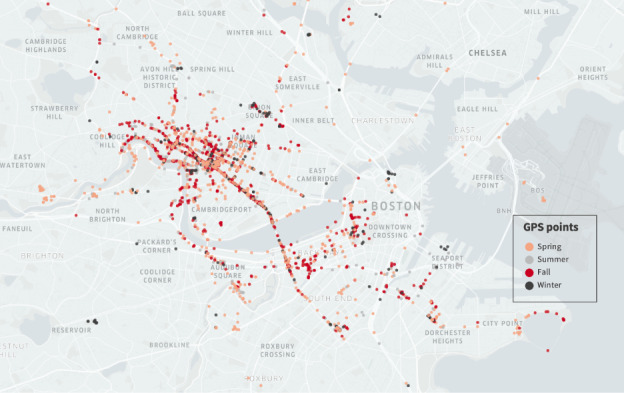
A visualization of GPS coordinates every 15 minutes across 4 seasons within the 1-year sampling period.

Table 3 presents smartphone-based GPS and accelerometer data collection compliance statistics at the participant day level based on total potential data collection days. We found that 40.38% (351,520/870,525) of observation days with GPS data and 40.02% (348,394/870,525) of observation days with accelerometer data were valid using a commonly applied criterion (≥10 valid hours). Broken down by phone OS, compliance with GPS and accelerometer data collection was nearly identical for data collected via Android and iOS devices at the participant day level (90,410/234,695, 38.52% vs 259,838/633,640, 41.01% for GPS data and 91,960/234,695, 39.18% vs 255,333/633,640, 40.3% for accelerometer data), although missing rates (ie, days with zero data) were lower for iOS devices than for Android devices for both data streams (291,756/633,640, 46.04% vs 134,395/234,695, 57.26% for GPS data and 290,141/633,640, 45.79% vs 118,368/234,695, 50.43% for accelerometer data). Figure 1 illustrates the GPS coordinates every 15 minutes over a 12-month period based on data collected from a test participant during protocol development.

Compliance rates at the participant day level were higher when calculated based on participant follow-up periods excluding attrition days after the Beiwe app was deleted from the participants’ phones (351,520/511,161, 68.77% vs 351,520/870,525, 40.38% for GPS data and 348,394/511,161, 68.16% vs 348,394/870,525, 40% for accelerometer data), suggesting that a substantial portion of our noncompliance was due to deletion of the app from participants’ phones before the end of the follow-up periods (participants might choose to unenroll from the study earlier and stop providing data) rather than to other possible reasons, such as a dead phone battery or app malfunction. Details of compliance calculated based on participant follow-up periods and the breakdown of rates by phone OS are provided in Multimedia Appendix 7. As a sensitivity analysis, we also compared participant day–level compliance for smartphone-based GPS and accelerometer data using a 10-hour cutoff and a more stringent 20-hour cutoff (Multimedia Appendix 8). The results indicate a substantial decrease in valid days (from 351,520/870,525, 40.38% to 99,694/870,525, 11.45% for GPS data and from 348,394/870,525, 40.02% to 38,613/870,525, 4.44% for accelerometer data).

Table 4 shows participant-level compliance with the smartphone GPS and accelerometer data collection using a 10-hour cutoff. GPS data showed that 39.52% (946/2394) of the participants had “excellent” compliance (≥75% of data collection days out of the total potential days were valid) and 11.95% (286/2394) had “good” compliance (between 50% and 75% of data collection days out of the total potential days were valid). For accelerometer data, 42.65% (1021/2394) of the participants had “excellent” compliance, and 10.61% (254/2394) had “good” compliance. The compliance proportions also varied by phone OS. For both the GPS and accelerometer data, Android users had a lower “excellent” or “good” compliance than iOS users (281/645, 43.6% vs 946/1743, 54.27% for GPS data and 317/645, 49.2% vs 953/1743, 54.68% for accelerometer data).

**Table 3:** Smartphone GPS and accelerometer data collection compliance at the participant day level by phone operating system (OS) for the Beiwe Smartphone Substudy of the Nurses’ Health Study 3 and Growing Up Today Study (N=870,525 total potential data collection days).

		Overall, n (%)	By phone OS, n (%)		
			Android (n=234,695)	iOS (n=633,640)	Both^a^ (n=2190)
**Participant day–level compliance rates—GPS data**					
	Valid days^b^	351,520 (40.38)	90,410 (38.52)	259,838 (41.01)	1272 (58.08)
	Invalid days	92,270 (10.6)	9890 (4.21)	82,046 (12.95)	334 (15.25)
	Noncollection days^c^	426,735 (49.02)	134,395 (57.26)	291,756 (46.04)	584 (26.67)
**Participant day–level compliance rates—accelerometer data**					
	Valid days^b^	348,394 (40.02)	91,960 (39.18)	255,333 (40.3)	1101 (50.27)
	Invalid days	113,090 (12.99)	24,367 (10.38)	88,166 (13.91)	557 (25.43)
	Noncollection days^c^	409,041 (46.99)	118,368 (50.43)	290,141 (45.79)	532 (24.29)

^a^Some participants switched smartphones during the 1-year data collection period, which resulted in a different OS.

^b^A valid day was defined as one with at least 600 valid minutes (10 valid hours) per day for GPS and accelerometer data. The participant day–level compliance was calculated as the percentage of valid days out of the total potential data collection days. Potential data collection days for each participant were defined as 365 days from the date the participant registered on the app.

^c^Missing days were defined as days without data. In this study, the primary reason for missing days was attrition (ie, the app was deleted from a participant’s phone before the 1-year study completion date because the participant unenrolled from the substudy). Another reason was sensor noncollection, which could be due to a participant forgetting to charge their phone or disabling the GPS or a major update from the OS causing the Beiwe app to malfunction temporarily, among other reasons.

**Table 4:** Smartphone GPS and accelerometer data collection compliance at the participant level by phone operating system (OS) for the Beiwe Smartphone Substudy of the Nurses’ Health Study 3 and Growing Up Today Study (N=2394).

		Overall, n (%)	By phone OS, n (%)		
			Android (n=645)	iOS (n=1743)	Both^a^ (n=6)
**Compliance rates** ^b^ **—GPS data**					
	Excellent (≥75%)	946 (39.52)	172 (26.67)	769 (44.12)	5 (83.33)
	Good (approximately 50%-75%)	286 (11.95)	109 (16.9)	177 (10.16)	0 (0)
	Fair (approximately 25%-50%)	328 (13.7)	94 (14.57)	234 (13.42)	0 (0)
	Poor (≤10%)	834 (34.84)	270 (41.86)	563 (32.3)	1 (16.67)
**Compliance rates** ^b^ **—accelerometer data**					
	Excellent (≥75%)	1021 (42.65)	241 (37.36)	775 (44.46)	5 (83.33)
	Good (approximately 50%-75%)	254 (10.61)	76 (11.78)	178 (10.21)	0 (0)
	Fair (approximately 25%-50%)	311 (12.99)	79 (12.25)	232 (13.31)	0 (0)
	Poor (≤10%)	808 (33.75)	249 (38.60	558 (32.01)	1 (16.67)

^a^Some participants switched smartphones during the 1-year data collection period, which resulted in a different OS.

^b^Compliance rates were defined as the percentage of valid days out of the potential data collection days for each participant. Potential data collection days for each participant were defined as the number of days between the date on which the participant registered on the app and the data download date (ie, November 1, 2022) selected for this study (if they had not yet completed a year of sampling) or the study end date of each participant (if they had completed a year of sampling). A valid day was defined as a day with ≥10 valid hours (600 valid minutes) of data records.

#### Survey Data

At the conclusion of the substudy, 87.72% (2100/2394) of the participants had completed 23,682 surveys. The average number of surveys completed per participant was 11 (SD 11; median 7) out of a total of 36 potential surveys that the participants were asked to complete. On average, Android users completed more surveys than iOS users (16, SD 13 vs 9, SD 10). A total of 41.62% (874/2100) of the participants completed at least 25% (10/37) of the surveys sent, 25.86% (543/2100) completed at least 50% (19/37) of the surveys sent, and 11.52% (242/2100) completed at least 75% (28/37) of the surveys sent. The average time to complete a survey since the participant opened it on the app was 53 (SD 197; median 21) seconds, which varied based on the number of questions in each survey. Table 5 shows the response rate for each of the substudy surveys sent to participants from the beginning to the end of the study except for the “pets” survey, which was administered at baseline and had a response rate of 82.71% (1980/2394). The average response rate for all the other surveys was 36% (SD 16%; median 41%), and the rate steadily declined after 1 year since the substudy began. Looking at the survey topics, 2 surveys on mental health had the lowest average response rates (average of 37% for “emotions” among 6 surveys administered and average of 38% for “stress/enjoyment” among 6 surveys administered), whereas all surveys on food intake (“fruits,” “nuts and dairy,” “beverages,” “meats,” and “vegetables”) had the highest response rates (>42% in all cases).

**Table 5:** Survey response rates for the Beiwe Smartphone Substudy of the Nurses’ Health Study 3 and Growing Up Today Study. Participants also completed the pet survey at the time of Beiwe app registration, with a response rate of 82.71% (1980/2394).

Survey date	Survey name	Response rate (%)	Survey submitted, n	Survey sent, N
August 5, 2021	Emotion	26.67	44	165
August 15, 2021	Stress and enjoyment	36.57	132	361
August 25, 2021	Physical activity	48.1	203	422
September 4, 2021	Green space	47.55	233	490
September 14, 2021	Sleep	51.22	273	533
September 26, 2021	Sitting	51.49	346	672
October 5, 2021	Emotion	79.83	562	704
October 15, 2021	Stress and enjoyment	52.04	433	832
October 25, 2021	Physical activity	50.88	461	906
November 4, 2021	Fruit	52.53	519	988
November 14, 2021	Vegetable	46.96	509	1084
November 24, 2021	Green space	52.1	595	1142
December 7, 2021	Sleep	45.36	562	1239
December 14, 2021	Sitting	47.95	597	1245
December 24, 2021	Emotion	18.97	233	1228
January 13, 2022	Stress and enjoyment	47.19	587	1244
January 13, 2022	Beverage	47.95	584	1218
January 22, 2022	Meat	47.88	588	1228
February 2, 2022	Green space	47.3	623	1317
February 13, 2022	Physical activity	47.33	629	1329
February 2, 2022	Sleep	47.76	629	1317
March 4, 2022	Sitting	45.25	590	1304
March 14, 2022	Nut dairy	46.69	648	1388
March 24, 2022	Fruit	44.7	624	1396
April 3, 2022	Vegetable	42.77	589	1377
April 13, 2022	Emotion	47.79	637	1333
April 23, 2022	Stress and enjoyment	52.7	703	1334
May 3, 2022	Physical activity	42.16	567	1345
May 13, 2022	Green space	43.08	607	1409
May 23, 2022	Sleep	45.67	649	1421
June 2, 2022	Sitting	46.84	667	1424
June 12, 2022	Nut dairy	43.97	616	1401
June 22, 2022	Beverage	42.56	589	1384
July 2, 2022	Meat	41.69	567	1360
July 12, 2022	Fruit	41.43	556	1342
July 22, 2022	Vegetable	42.50	561	1320
August 4, 2022	Emotion	32.21	411	1276
August 14, 2022	Stress and enjoyment	11.93	142	1190
August 26, 2022	Physical activity	37.49	391	1043
September 6, 2022	Green space	33.44	327	978
September 16, 2022	Sleep	21.21	183	863
September 26, 2022	Sitting	19.04	155	814
October 6, 2022	Emotion	24.97	187	749
October 16, 2022	Stress and enjoyment	26.63	184	691
October 26, 2022	Physical activity	24.47	149	609
November 5, 2022	Fruit	24.68	134	543
November 15, 2022	Vegetable	25.1	122	486
November 25, 2022	Green space	26.92	112	416
December 5, 2022	Sleep	25.48	93	365
December 15, 2022	Sitting	24.53	78	318
December 25, 2022	Emotion	24.91	72	289
January 4, 2023	Stress and enjoyment	24.34	65	267
January 14, 2023	Beverage	26.32	65	247
January 24, 2023	Meat	24.48	59	241
February 4, 2023	Green space	23.11	52	225
February 14, 2023	Physical activity	24.44	44	180
February 24, 2023	Sleep	25	41	164
March 6, 2023	Sitting	23.57	37	157
March 15, 2023	Nut dairy	11.81	17	144
March 27, 2023	Fruit	20	20	100
April 7, 2023	Vegetable	16.85	15	89
April 17, 2023	Emotion	3.7	3	81
April 27, 2023	Stress and enjoyment	17.95	14	78
May 8, 2023	Physical activity	19.05	12	63
May 18, 2023	Green space	10	5	50
June 1, 2023	Sleep	10	1	10

### Preliminary Summary Statistics of Participant Mobility, Walking Behavior, and Chronic Disease Drivers

#### GPS and Accelerometer Data

Table 6 presents the preliminary summary statistics of participant mobility and walking behavior metrics obtained from processed GPS and accelerometer data. After applying the missing data imputation algorithm [37], we found that participants spent an average of 14.6 (SD 7.5; median 15.1) hours per day at home and 1.6 (SD 1.6; median 1.2) hours per day on trips on 439,907 participant days. In addition, the median distance that participants traveled per day was 26.6 (IQR 7.9-61.6) km, and the median number of locations visited was 3. Time spent at home and on trips, distance traveled, and the number of places visited differed only slightly by phone OS.

**Figure 2:**
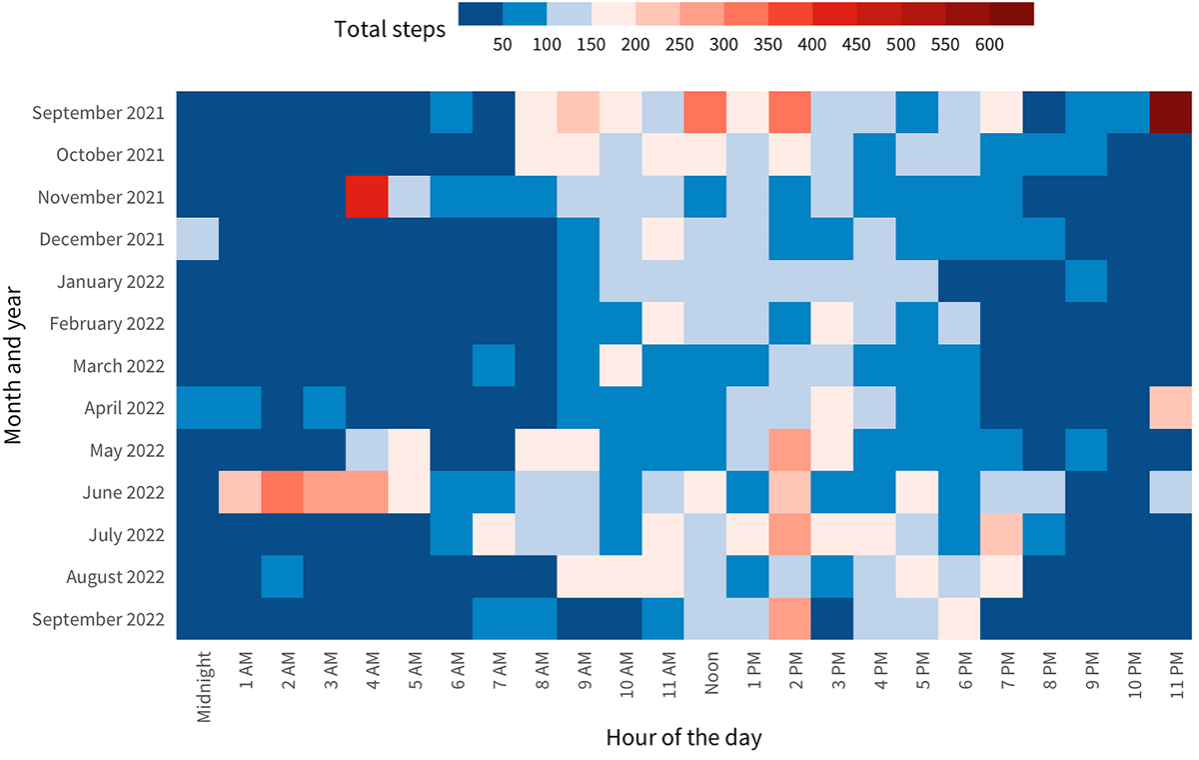
Hourly walking steps by month over 12 months using the accelerometer data of a randomly selected participant.

For walking behavior, we sampled an average of 1046 (SD 1029; median 761) steps per day and 9.4 (SD 9.2; median 6.9) minutes of walking per day within the 8-hour daily observation periods (the app was configured so that the smartphone collected 30 seconds of accelerometer data every 90 seconds to conserve smartphone battery) over 456,485 participant days. When comparing phone OS, iOS users had more daily steps and minutes of walking than Android users (1125 vs 805 steps and 10.2 vs 7.3 minutes). Figure 2 shows hourly walking steps per month over a 12-month period based on data collected during protocol development from a randomly selected participant.

**Table 6:** Preliminary mobility and walking behavior metrics derived from processed smartphone GPS and accelerometer data from the Beiwe Smartphone Substudy of the Nurses’ Health Study 3 and Growing Up Today Study.

				Overall		By phone OS^a^						
						Android			iOS		Both^b^	
**Participant mobility^c^**												
	**Time spent at home (hours)**											
		Mean (SD)	14.6 (7.5)		14.6 (7.8)			14.6 (7.4)		12.4 (7.9)		
		Median (IQR)	15.1 (10.5-21.0)		15.1 (10.2-21.5)			15.1 (10.5-20.8)		12.4 (6.6-19.2)		
	**Time spent on trips (hours)**											
		Mean (SD)	1.6 (1.6)		1.4 (1.3)			1.6 (1.6)		1.8 (1.8)		
		Median (IQR)	1.2 (0.6-2.0)		1.2 (0.7-1.8)			1.2 (0.6-2.1)		1.3 (0.7-2.3)		
	**Total distance traveled per day (km)**											
		Mean (SD)	84.8 (379.0)		80.3 (315.4)			86.0 (395.7)		132.3 (408.7)		
		Median (IQR)	26.6 (7.9-61.6)		27.0 (7.7-65.7)			26.4 (7.9-60.3)		35.8 (6.4-84.3)		
	**Number of significant^d^ locations visited per day**											
		Mean (SD)	3.4 (2.1)		3.1 (2.0)			3.5 (2.2)		3.9 (2.3)		
		Median (IQR)	3.0 (2.0-4.0)		3.0 (2.0-4.0)			3.0 (2.0-5.0)		4.0 (2.0-5.0)		
**Participant walking behaviors^e^**												
	**Total daily walking minutes^f^**											
		Mean (SD)	9.4 (9.2)		7.3 (8.4)		10.2 (9.4)					10.5 (11.6)
		Median (IQR)	6.9 (3.1-12.9)		4.5 (1.6-9.9)		7.7 (3.8-13.7)					8.2 (4.1-13.5)
	**Total daily step counts^f^**											
		Mean (SD)	1045.6 (1029.4)		805.2 (940.6)		1125.0 (1044.0)					1136.1 (1258.0)
		Median (IQR)	760.7 (341.3-1421.6)		492.5 (175.1-1092.4)		847.2 (416.5-1513.7)					886.2 (449.3-1455.8)

^a^OS: operating system.

^b^Some participants switched smartphones during the 1-year data collection period, which resulted in a different OS.

^c^Overall: n=439,907; Android: n=99,699; iOS: n=338,604; and both: n=1605.

^d^Significant locations were defined as distinct locations visited that were at least 50 meters away and whose visit lasted at least 15 minutes.

^e^Overall: n=456,485; Android: n=113,399; iOS: n=341,443; and both: n=1512.

^f^The walking behavior metrics shown reflect only what the smartphone accelerometer observed during the day when it was on (the accelerometer was configured to be on for 30 seconds during the 90-second cycle to conserve the smartphone battery). If we extrapolate the time at a ratio of 1:3 (ie, 8 out of 24 hours per day), we expect approximately 27 minutes of daily walking time and approximately 3000 steps per day recorded by the smartphone.

#### Survey Data

**Figure 3:**
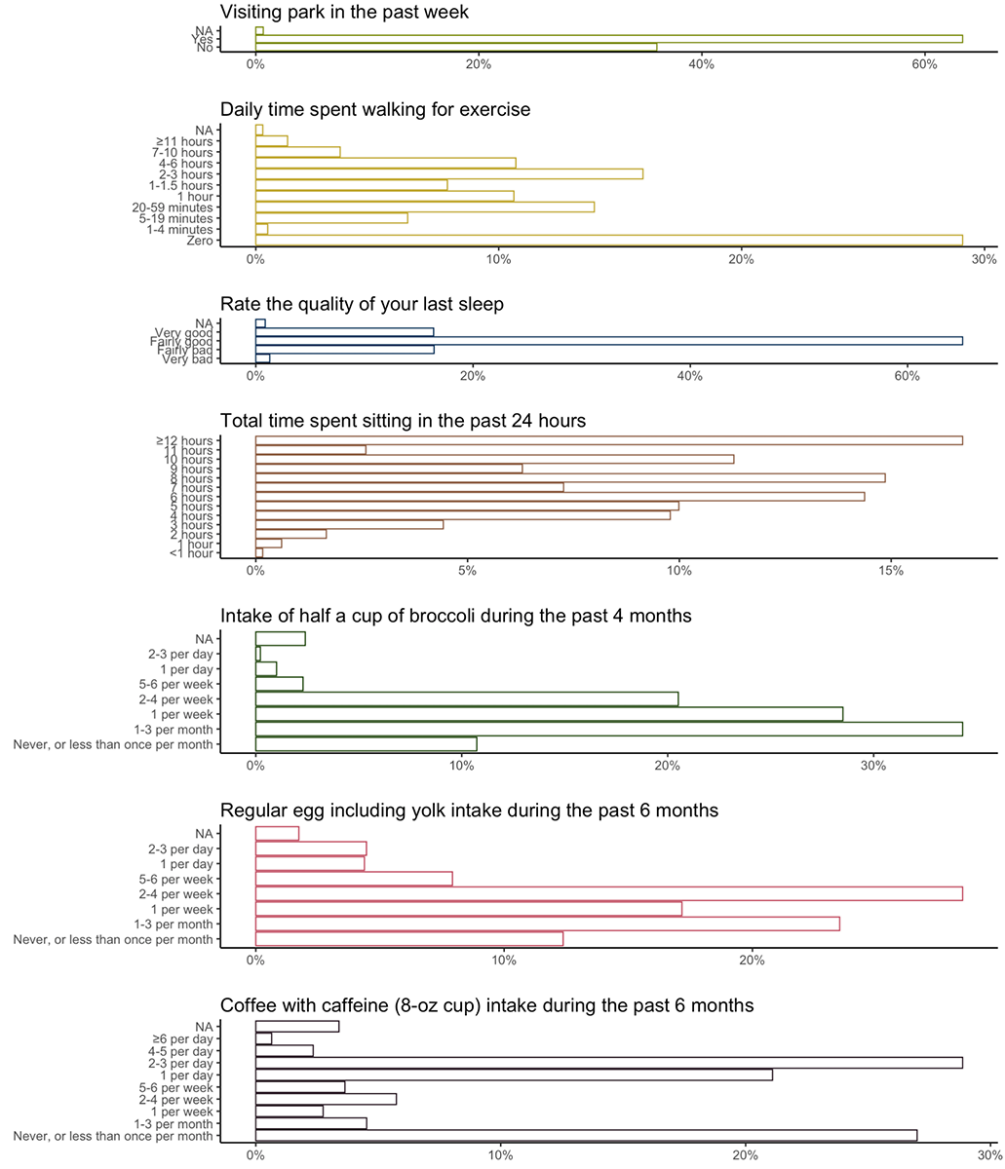
The distribution of responses to selected questions on behavioral factors in the smartphone-based surveys for the Beiwe Smartphone Substudy of the Nurses’ Health Study 3 and Growing Up Today Study. NA: not applicable.

**Figure 4:**
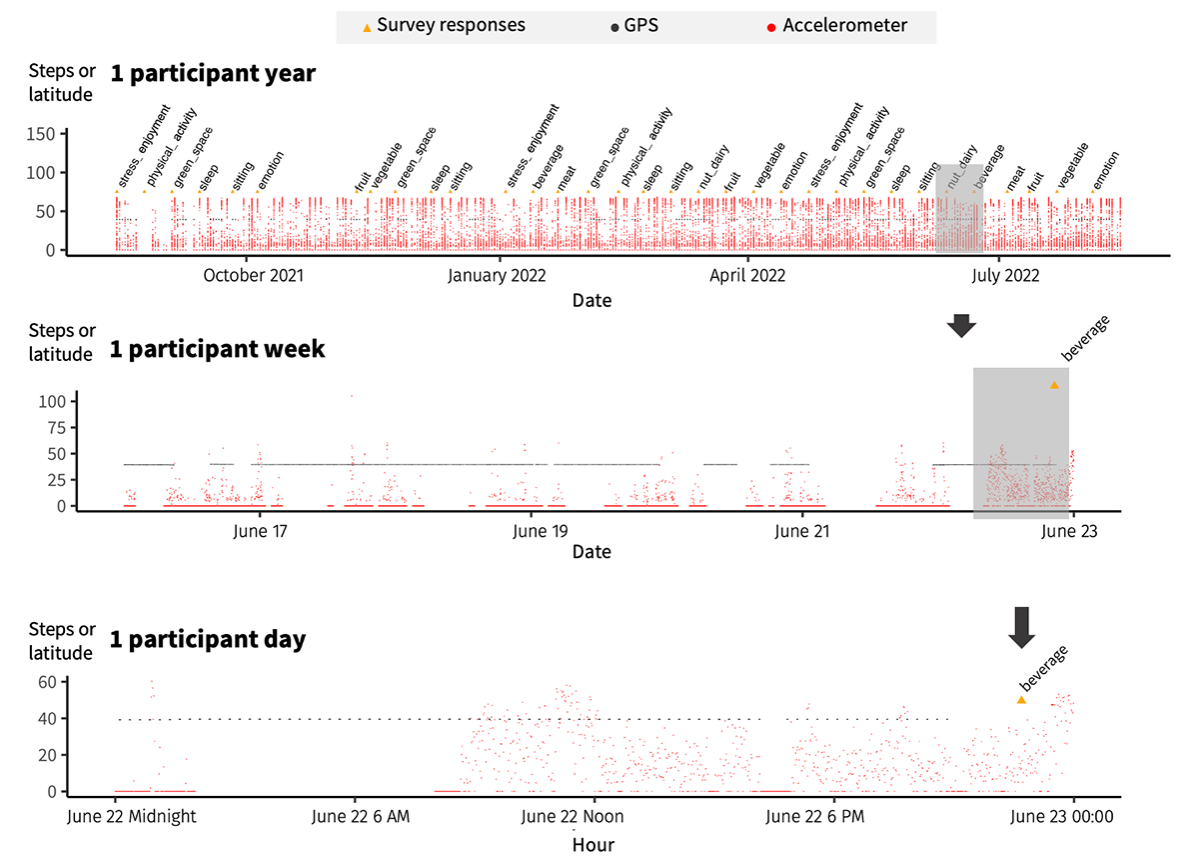
A visualization of 3 streams (GPS, accelerometer, and survey) of digital phenotyping data from a participant in the Beiwe substudy at the participant day, participant week, and participant year levels, with red dots representing the step count at each minute, black dots representing latitude as a proxy for GPS availability at each minute, and orange dots representing each submitted survey.

In addition to walking behavior and mobility, we obtained the prevalence of important environmental and behavioral factors from 22,783 survey responses, including park visits, leisure-time walking, fruit and vegetable consumption, timing and duration of sleep, and daily sitting time. Figure 3 shows the distribution of the responses to a selected list of survey questions. For example, regarding participants’ physical activity and sedentary behavior, a little less than two-thirds of the survey submissions (1609/2539, 63.37%) reported visiting a park in the previous week, approximately 50% (1222/2447) reported walking for at least 1 hour for exercise in the previous week, and >50% (1274/2463, 51.73%) reported sitting for at least 8 hours in the previous day. In addition, more than four-fifths of the survey submissions (1970/2419, 81.44%) reported having a fairly good or very good quality of sleep. To cite some examples of food intake, 86.86% (1554/1789) of survey submissions reported eating half a cup of broccoli at least 1 to 3 times per month in the previous 4 months, 85.89% (1041/1212) reported eating eggs (including egg yolks) at least 1 to 3 times per month in the previous 6 months, and slightly less than 70% (861/1237, 69.6%) reported drinking caffeinated coffee (8-oz cup) at least 1 to 3 times per month in the previous 6 months. Figure 4 illustrates an example survey schedule alongside the GPS and accelerometer data for a substudy participant at the participant day, participant week, and participant year levels.

## Discussion

### Principal Findings

Physical inactivity, prolonged sitting, and poor dietary habits are important behavioral risk factors for chronic diseases that are prevalent in the United States and can be influenced by environmental factors. Simultaneously, mHealth technology such as digital phenotyping offers unique insights into individuals’ behavioral and physiological traits, including geographic locations, movements, and lifestyle choices, which can be used to derive environmental exposures and behavioral drivers of chronic diseases. In this study, we integrated smartphone-based digital phenotyping into 2 well-established nationwide prospective cohorts, the NHS3 and GUTS, and described our approach to manage and analyze a vast amount of digital phenotyping data collected at a high spatial and temporal resolution. Our initial assessment of adherence to smartphone-based data collection protocols over 1 year indicates that adherence rates in our study were either higher or similar to those of most previous studies with much shorter follow-up periods and smaller sample sizes. In addition, we presented preliminary summary statistics on participant mobility, walking behavior, and potential environmental and behavioral drivers of chronic diseases derived from 1 year of smartphone data. These location-stamped behavior data can be linked to environmental datasets (eg, air pollution, temperature, and greenness) to examine associations between momentary (eg, greenness) or transient (eg, extreme heat) environmental exposures and health behaviors (eg, physical activity) or other outcomes (eg, mental health) in this population. Furthermore, the participants in this study were members of the ongoing NHS3 and GUTS prospective cohorts, so there is rich covariate history from previous questionnaires to derive insights on how demographics, comorbidities, and other health behaviors affect exposures and behaviors. Moreover, these participants will be followed for decades for chronic disease incidence, so these digital phenotyping data can serve as baseline exposure and behavior data for future analyses.

Our low-burden and cost-effective digital phenotyping data collection protocols achieved overall device data completeness and survey response rates (ie, valid data collection hours per day, percentage of participants meeting the compliance cutoff, and percentage of surveys answered) that were on par with, if not higher than, other recent smartphone-based digital phenotyping studies [25,28,30,39-42] despite the fact that most of these studies had much smaller sample sizes and shorter data collection lengths. According to a recent review by Lee et al [43] of studies that used digital phenotyping to understand health-related outcomes, the sample sizes for such studies were generally <100, and the duration was usually between 10 and 14 days. There were a few studies with larger samples [30,31,44-49], for example, the Interventions, Research, and Action in Cities Team cohort study designed by Kestens et al [31], which applied “Ethica” (another popular digital phenotyping platform) to follow approximately 2000 participants within the 5-year period to investigate changes in health behavior and mental health outcomes before and after the changes made to the built environment [30,31]. However, most of these studies had shorter sampling periods of 2 to 6 weeks (eg, 30 consecutive days for the aforementioned study), unlike our study, where more than three-quarters of participants provided data for >6 weeks. However, despite our longer-than-usual data collection length, we also found that many participants deleted the app before the end of the 1-year follow-up period. Unfortunately, we did not conduct a feedback survey at the end of the substudy to ask about the reasons for dropping out. This could have helped us to gather user experience data and understand participants’ willingness to provide data over a long follow-up period (1 year in our case). We hope to do this in future studies.

Our findings on differences in device compliance according to phone OS contribute to the growing literature on this topic. Specifically, we found that iOS users in the study had lower noncollection rates and higher participant-level compliance than Android users for both GPS and accelerometer data. These findings are partially consistent with those of Kiang et al [40], who examined GPS and accelerometer data noncollection rates in 6 digital phenotyping studies using the Beiwe platform and found that iOS users had lower GPS data noncollection rates than Android users. Kiang et al [40] further investigated whether GPS and accelerometer data noncollection rates differed by sociodemographic characteristics as differences in participant demographics by iPhone and Android phones have recently been reported [50]; however, they ultimately reported null results. To investigate this issue in our study, we compared the sociodemographic characteristics of the 2 groups (Multimedia Appendix 9). In contrast to Kiang et al [40], we found that iOS users in our study in general had higher household incomes than Android users. Therefore, we speculate that the differences in compliance by phone OS in our study may be due to differences in the socioeconomic status (SES) of the participants. We suggest that future studies with more diverse populations are needed to further investigate whether participants’ SES at the individual or area level could account for the difference in adherence to smartphone data collection according to phone OS.

Regarding the preliminary statistics of participant mobility and walking behavior derived from GPS and accelerometer data, we found that the values were lower than those in previous studies using wearable devices. For example, we found that the average number of steps per day was much lower than that in another NHS3 study [39]. This discrepancy is due to difference in wear patterns between wearables (ie, Fitbit) and the smartphone-only design used in our study, as well as the fact that our protocol samples GPS and accelerometer data noncontinuously by design [51]. A recent study by Straczkiewicz et al [52] found similar discrepancies in walking outcomes by comparing step counts obtained from smartphone-based accelerometers and Fitbit devices, which is consistent with our findings. It is important to point out that, if we extrapolate the total number of steps to the 24-hour period (average of 996 steps over 400,000 participant days at 8 hours per day), we estimate that we would obtain approximately 3000 steps per day. This does not consider that approximately 70% of the participants in our sample (1703/2394, 71.13%) were nurses who may not have always had a smartphone with them during their daily activities. In addition, in this case, the extrapolated number was closer to the lower end of the average number of steps taken by American adults, as the latest study of smartphone-based physical activity data shows [53]. Despite the aforementioned limitations, the method we used to quantify walking activity was found to be a reliable approach for identifying walking bouts and deriving step counts and walking minutes [32]. In the future, we plan to compare smartphone surveys to objective smartphone-based accelerometer measurements to further explore this topic.

The high spatial and temporal resolution of GPS and accelerometer data allowed us to examine variations (minute by minute and daily) in environmental exposure and physical activity within each participant (see an example in Figure 4), a resolution that conventional large epidemiological studies often fail to achieve. Our data collection over a year offers an advantage over the short duration (typically 7-14 days) of data collection in many intensive longitudinal studies, which often rely on GPS devices and accelerometers to collect location and movement data [29] and place a greater burden on participants. Smartphone-based location data may be particularly advantageous over dedicated GPS devices at the day or survey level as recent studies have reported small differences between activity space measures derived from location data collected by these 2 types of devices [26]. In addition, most people have their smartphones with them throughout the day and charge their phone every night [54]. We believe that our data have enormous potential to advance the field of environmental exposure assessment and behavioral measurement in cohort studies and provide new insights into how the environment and behavioral factors drive chronic disease outcomes. In the following paragraphs, we discuss potential applications of our data.

To start, with minute-level GPS data collected from >2400 participants across a 12-month period (see Figure 1 for an example illustration of the richness of our GPS data), our study can assess and compare personalized exposures to various environmental characteristics (eg, air pollution, green spaces, walkability, and noise) at distinct temporal resolutions (eg, 7 days, 1 month, 3 months, and the entire year) accounting for exposure as participants move through time and space and across a year. This can then be used to examine how these smartphone-based measurements compare to traditional residential-based environmental and self-reported behavioral measurements. For example, we can compare participants’ greenness exposure in their residential neighborhoods with exposure in their activity spaces aggregated at different temporal scales [21]. The results of this study will enable us to develop regression calibration methods that can be applied to correct residential-based measures of environmental exposure and self-reported physical activity in the larger NHS3 and GUTS cohorts. This may mitigate the potential bias and misclassification that could affect associations between these exposures and health behaviors or outcomes.

In addition, the highly personalized exposure metrics give us a better position to answer important research questions in the field of activity space–based health research, such as the number of days of GPS monitoring required to adequately capture an individual’s environmental exposure or behavioral patterns [55]. In addition, the minute-by-minute walking data of our participants allowed us to examine their physical activity patterns at daily or diurnal levels (see Figure 2 for an example of the high-resolution step data over a year). Both examples illustrate how our data are better suited for determining the effects of environmental and behavioral drivers of many chronic diseases at both the intraindividual (eg, days within a participant) and interindividual levels.

More importantly, the 3 streams of smartphone-based digital phenotyping data collected in our study can be linked at different spatial and temporal resolutions to quantify the relationships between environmental factors and health behaviors. For example, 2 recent studies reported nonlinear minute-level relationships among greenness exposure, walkability, and physical activity outcomes [21,56]. However, both studies spanned only 7 days, which may not fully capture the typical environmental exposures in an individual’s activity space and physical activity behavior. In this regard, our study adds a valuable longitudinal perspective that may capture routine behaviors over long periods and complements existing intensive longitudinal studies that observe participants for between 7 and 14 days without compromising data resolution. Finally, we could examine noncontemporaneous effects of environmental exposure, such as time-lagged responses, on health behaviors [14], for example, whether environmental exposure from the previous day affects self-reported stress scores from surveys. Investigating the association with different temporalities (eg, time-lagged relationships in the previous example or the same-day relationship), of environmental exposure is a critical step toward understanding the various mechanisms that may influence health-related outcomes [44].

### Challenges, Limitations, and Strengths

We encountered 2 major challenges in conducting this study. First, the steep learning curve for exploring, setting up, and fine-tuning an optimized computing infrastructure for managing, processing, and analyzing the vast amount of digital phenotyping data from smartphones can be an obstacle for researchers who may lack the technical skills required to manage this quantity of data. In our study, we adopted a cloud-based approach using AWS for data storage and processing. Other studies may benefit from other computing infrastructures depending on their design, data characteristics, and data processing budgets. As the number of digital phenotyping studies increases, further discussion on the advantages and disadvantages of each approach is required. Second, privacy concerns remained. The personally identifiable information collected in this study, such as high-resolution location data collected through GPS, may reveal sensitive information, such as the participants’ home and work location and daily travel activities. In this study, we addressed this issue by encrypting data during transmission and storing the processed data in an institutional review board–approved AWS structure. We also restricted data access so that the personally identifiable information was only accessed when needed and was never shared with other parties.

Our study had certain limitations that should be considered. First, the participants were selected from 2 prospective cohort studies, which primarily consisted of White female individuals of a relatively high SES. Therefore, it is important to note that our findings may not be applicable to other populations. In addition, the ratio of male to female individuals invited to participate in the substudy was slightly higher than in the group that we did not invite because we started recruiting male participants in later data collection cycles in the NHS3, which may result in a slightly less representative sample of the whole cohort. Second, the GPS and accelerometer data missingness may affect the representativeness of the data collected (eg, GPS trajectories recorded vs actual trajectories). Much of the missing data could be due to our study design as we configured our Beiwe app to collect data in preprogrammed on-off cycles. In addition, a large proportion of our participants were nurses who may not be permitted to use their personal smartphones during work hours. This may have contributed to lower adherence to the study protocol. Unfortunately, we did not collect information on whether participants were allowed to carry their smartphones during working hours; therefore, we could not investigate how this affected our results. We also did not conduct a feedback survey at the end of the substudy to ask about the reasons for dropping out, which could have helped us gather user experience data and better understand participants’ willingness to provide data within a long follow-up period (1 year in our case). To reduce the missingness of smartphone-based survey data, we shortened the questions we asked in each smartphone survey to minimize the overall risk of burdening participants. However, we did not conduct a feedback survey to allow participants to comment on the experience and content of the smartphone surveys; therefore, we were unable to evaluate the effectiveness of our strategy. Overall, the missingness in the aforementioned datasets may affect our ability to answer research questions related to associations between environmental exposures and health behaviors and their roles as potential drivers of chronic diseases [51]. Nevertheless, the data of our study are still substantially larger in volume than those from most previous mHealth studies.

Despite the challenges and limitations, our study is one of the largest digital phenotyping efforts conducted to date in terms of both the number of participants and duration of data collection. Setting up a cloud-based computing infrastructure that leverages the powerful capabilities of AWS proved cost-effective in handling the immense amount of smartphone-based digital phenotyping data that we received daily. This, together with the fact that the Beiwe app is an open-source research platform for smartphone-based high-throughput digital phenotyping that can be accessed worldwide [36], indicates that our approach is scalable and could prove particularly beneficial for future studies with similar goals. Most importantly, the high spatial and temporal resolution of the data from the GPS and accelerometers allows us to examine variations in environmental and behavioral risk factors at multiple spatial and temporal scales and explore the complex relationships between exposures and behaviors. This ultimately helps shed light on their roles as drivers of many chronic diseases that are prevalent in the United States.

### Conclusions

In summary, despite the relatively high dropout rates during the 1-year data collection period and the representativeness of our data due to noncontinuous data collection, our assessment of adherence to data collection protocols in the 1-year Beiwe Smartphone Substudy of the NHS3 and GUTS revealed an overall device data completeness and survey response rates comparable to, if not higher than, those of other current smartphone-based digital phenotyping studies with smaller participant numbers and much shorter data collection periods. As mHealth technologies grow in popularity and collect vast amounts of data, this substudy lays the groundwork for further development of techniques to extract meaningful information from the noise of the vast amount of streamed smartphone-based digital phenotyping data. We will also be able to assess the length of time required to develop reliable measures of activities. These highly granular spatially and temporally resolved mobility and health behavior data could complement the annual questionnaires in large epidemiological cohorts to provide new insights into the impact of mobility-based exposure to air pollutants, noise, and green spaces on physical inactivity, poor dietary habits, inadequate sleep, and mental well-being, all of which are important risk factors for chronic diseases.

## Appendix

Multimedia Appendix 1 CONSORT (Consolidated Standards of Reporting Trials) diagram of participant numbers at different recruitment stages of the Beiwe Smartphone Substudy of the Nurses’ Health Study 3 and Growing Up Today Study.

Multimedia Appendix 2 Screenshots of the customized Beiwe app used in the Beiwe Smartphone Substudy of the Nurses’ Health Study 3 and Growing Up Today Study.

Multimedia Appendix 3 A list of the questions asked in the 12 surveys of the Beiwe Smartphone Substudy of the Nurses’ Health Study 3 and Growing Up Today Study.

Multimedia Appendix 4 Demographic characteristics of active participants in the Nurses’ Health Study 3 and Growing Up Today Study cohorts at baseline who were invited or not invited to the substudy.

Multimedia Appendix 5 Demographic characteristics of participants in the Beiwe Smartphone Substudy of the Nurses’ Health Study 3 and Growing Up Today Study who provided at least 30, 60, and 90 days of data.

Multimedia Appendix 6 GPS and accelerometer data collection compliance for the Beiwe Smartphone Substudy of the Nurses’ Health Study 3 and Growing Up Today Study.

Multimedia Appendix 7 Smartphone GPS and accelerometer data collection compliance at the participant day level based on participant follow-up period by phone operating system for the Beiwe Smartphone Substudy of the Nurses’ Health Study 3 and Growing Up Today Study.

Multimedia Appendix 8 The participant day–level smartphone-based GPS and accelerometer data collection compliance according to 2 validity criteria.

Multimedia Appendix 9 Demographic characteristics of the participants in the Beiwe Smartphone Substudy of the Nurses’ Health Study 3 and Growing Up Today Study with the breakdown by phone operating system.

## Abbreviations

AWS: Amazon Web Services
GUTS: Growing Up Today Study
mHealth: mobile health
NHS3: Nurses’ Health Study 3
OS: operating system
SES: socioeconomic status

## Data Availability

The datasets generated during and analyzed during this study are available in the controlled-access repositories [57,58], except for personal identifying data such as names, addresses, smartphone location data, and contact information.
