# Supplementary material for: Measuring Environmental and Behavioral Drivers of Chronic Diseases Using Smartphone-Based Digital Phenotyping: Intensive Longitudinal Observational mHealth Substudy Embedded in 2 Prospective Cohorts of Adults
Source: JMIR Public Health Surveill. 2024 Oct 11;10:e55170. doi: 10.2196/55170 (PMC11512133; doi:10.2196/55170)
Supplement: Multimedia Appendix 1 [file publichealth_v10i1e55170_app1.docx]

| 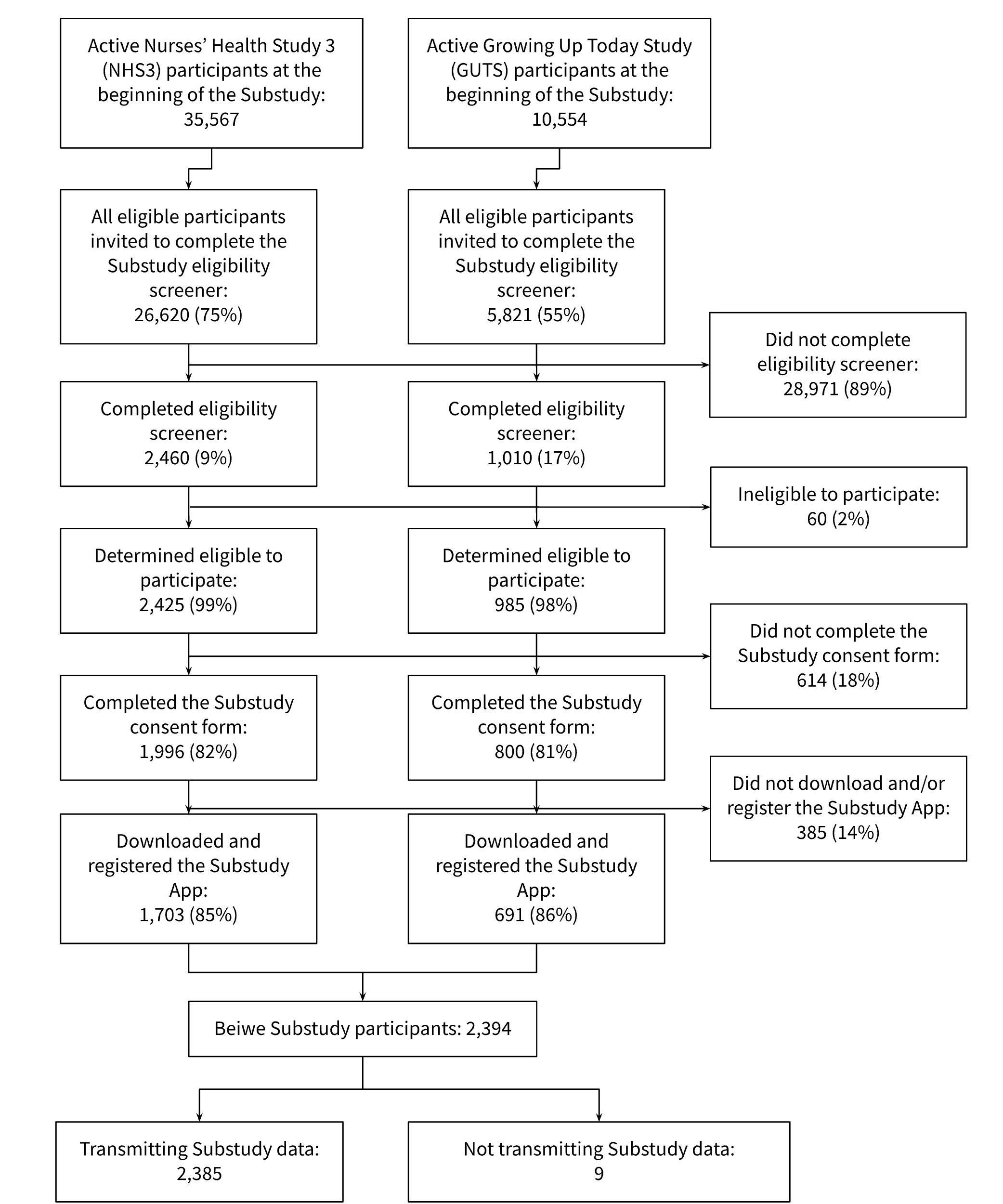 |
| --- |
|  |
| **Figure S1.** A CONSORT diagram of participation numbers at different recruitment stages of the Beiwe Smartphone Substudy of Nurses' Health Study 3 (NHS3) and Growing Up Today Study (GUTS). |
