# Supplementary material for: Measuring Environmental and Behavioral Drivers of Chronic Diseases Using Smartphone-Based Digital Phenotyping: Intensive Longitudinal Observational mHealth Substudy Embedded in 2 Prospective Cohorts of Adults
Source: JMIR Public Health Surveill. 2024 Oct 11;10:e55170. doi: 10.2196/55170 (PMC11512133; doi:10.2196/55170)
Supplement: Multimedia Appendix 2 [file publichealth_v10i1e55170_app2.docx]

| 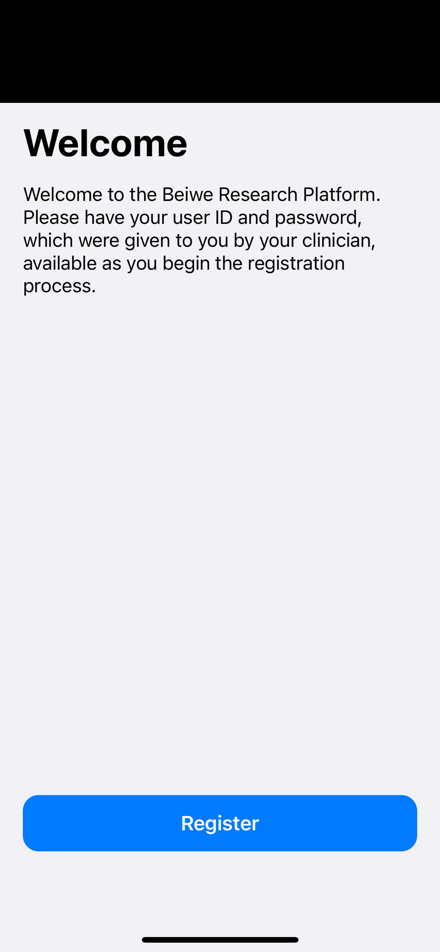 |  | 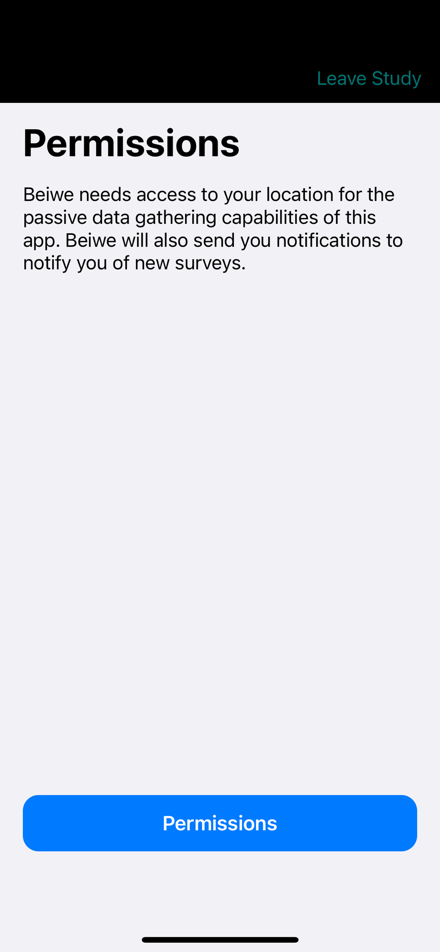 |  | 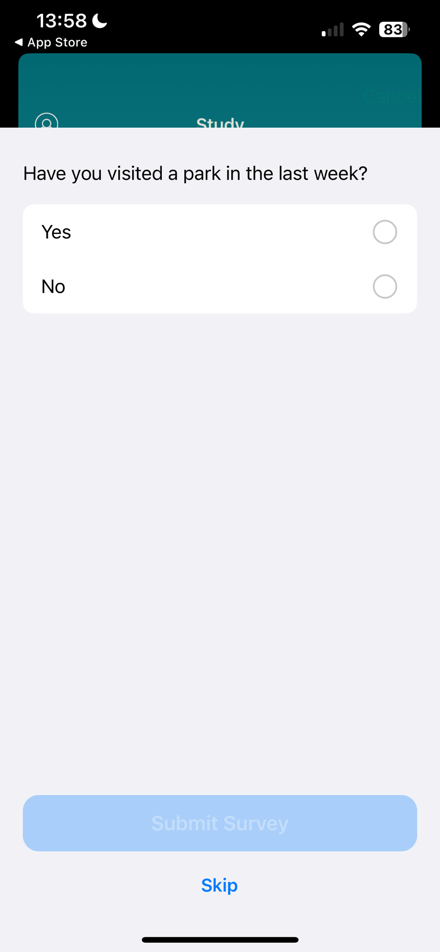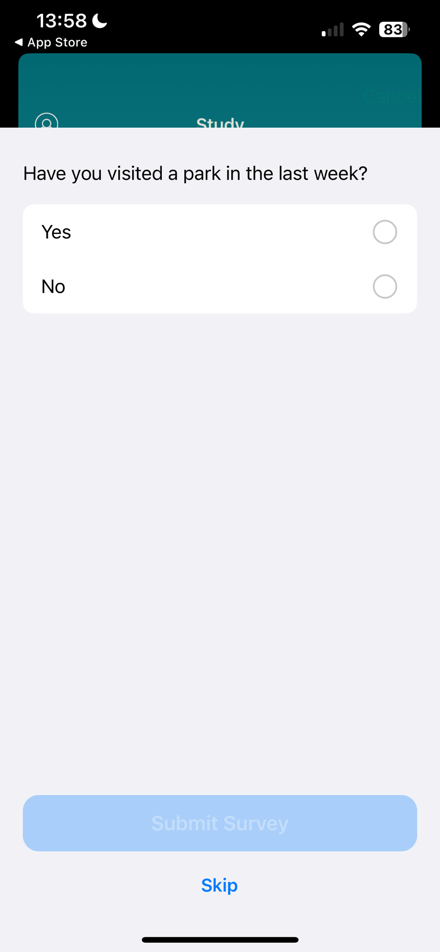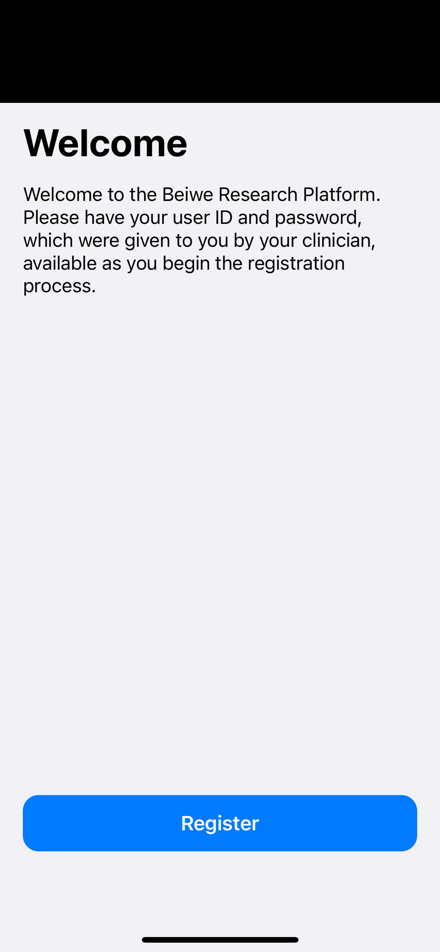 |
| --- | --- | --- | --- | --- |
|  | | | | |
| **Figure S2.** Screenshots of the customized Beiwe app used in the Beiwe Smartphone Substudy of Nurses’ Health Study 3 (NHS3) and Growing Up Today Study (GUTS). | | | | |
| Notes. **Left**: a “welcome screen” at initial registration; **Center**: a “request for permission" screen” after initial registration; **Right**: the screen when participants answer the first question of the “green space” survey. | | | | |
