# Supplementary material for: Measuring Environmental and Behavioral Drivers of Chronic Diseases Using Smartphone-Based Digital Phenotyping: Intensive Longitudinal Observational mHealth Substudy Embedded in 2 Prospective Cohorts of Adults
Source: JMIR Public Health Surveill. 2024 Oct 11;10:e55170. doi: 10.2196/55170 (PMC11512133; doi:10.2196/55170)
Supplement: Multimedia Appendix 3 [file publichealth_v10i1e55170_app3.docx]

| **Table S1.** A list of questions asked in the 12 surveys of the Beiwe Smartphone Substudy of Nurses' Health Study 3 (NHS3) and Growing Up Today Study (GUTS). |
| --- |

**Green Space Survey**

| **Variable Name** | **Corresponding Questions** | **Question Options** |
| --- | --- | --- |
| Starting Prompt: N/A | | Type: Radio Button |
| q1_visited_park | Have you visited a park in the last week? | Yes, No |
| q2_spent_time_in_nature | Have you spent time in nature in the last week? | Yes, No |

**Fruits Survey**

| **Variable Name** | **Corresponding Questions** | **Question Options** |
| --- | --- | --- |
| Starting Prompt: Please indicate how often you consumed the specified amount of each food during the past 4 months. | | Type: Radio Button |
| q1_raisin_or_grape | Raisins (1 oz. or small pack) or grapes (½ cup) | Never, or less than once per month , 1-3 per month , 1 per week , 2-4 per week , 5-6 per week , 1 per day , 2-3 per day , 4-5 per day , 6+ per day |
| q2_prune_or_dried_plum | Prunes or dried plums (6 prunes or ¼ cup) | Never, or less than once per month , 1-3 per month , 1 per week , 2-4 per week , 5-6 per week , 1 per day , 2-3 per day , 4-5 per day , 6+ per day |
| q3_juice_prune | Prune juice (small glass) | Never, or less than once per month , 1-3 per month , 1 per week , 2-4 per week , 5-6 per week , 1 per day , 2-3 per day , 4-5 per day , 6+ per day |
| q4_banana | Bananas(1) | Never, or less than once per month , 1-3 per month , 1 per week , 2-4 per week , 5-6 per week , 1 per day , 2-3 per day , 4-5 per day , 6+ per day |
| q5_cantaloupe | Cantaloupe (¼ melon) | Never, or less than once per month , 1-3 per month , 1 per week , 2-4 per week , 5-6 per week , 1 per day , 2-3 per day , 4-5 per day , 6+ per day |
| q6_avocado | Avocado (½ fruit or ½ cup) | Never, or less than once per month , 1-3 per month , 1 per week , 2-4 per week , 5-6 per week , 1 per day , 2-3 per day , 4-5 per day , 6+ per day |
| q7_apple_or_pear | Fresh apples or pears (1) | Never, or less than once per month , 1-3 per month , 1 per week , 2-4 per week , 5-6 per week , 1 per day , 2-3 per day , 4-5 per day , 6+ per day |
| q8_juice_or_cider_apple | Apple juice or cider (small glass) | Never, or less than once per month , 1-3 per month , 1 per week , 2-4 per week , 5-6 per week , 1 per day , 2-3 per day , 4-5 per day , 6+ per day |
| q9_orange | Oranges (1) | Never, or less than once per month , 1-3 per month , 1 per week , 2-4 per week , 5-6 per week , 1 per day , 2-3 per day , 4-5 per day , 6+ per day |
| q10_juice_regular_orange | Regular orange juice (not calcium fortified) (small glass) | Never, or less than once per month , 1-3 per month , 1 per week , 2-4 per week , 5-6 per week , 1 per day , 2-3 per day , 4-5 per day , 6+ per day |
| q11_juice_calc_orange | Orange juice, calcium fortified (small glass) | Never, or less than once per month , 1-3 per month , 1 per week , 2-4 per week , 5-6 per week , 1 per day , 2-3 per day , 4-5 per day , 6+ per day |
| q12_grapefruit | Grapefruit (½) or grapefruit juice (small glass) | Never, or less than once per month , 1-3 per month , 1 per week , 2-4 per week , 5-6 per week , 1 per day , 2-3 per day , 4-5 per day , 6+ per day |
| q13_juice_other | Other fruit juices (small glass) | Never, or less than once per month , 1-3 per month , 1 per week , 2-4 per week , 5-6 per week , 1 per day , 2-3 per day , 4-5 per day , 6+ per day |
| q14_strawberry | Strawberries, fresh, frozen, or canned (½ cup) | Never, or less than once per month , 1-3 per month , 1 per week , 2-4 per week , 5-6 per week , 1 per day , 2-3 per day , 4-5 per day , 6+ per day |
| q15_blueberry | Blueberries, fresh, frozen, or canned (½ cup) | Never, or less than once per month , 1-3 per month , 1 per week , 2-4 per week , 5-6 per week , 1 per day , 2-3 per day , 4-5 per day , 6+ per day |
| q16_peach_plum | Peaches or plums (1 fresh or ½ cup canned) | Never, or less than once per month , 1-3 per month , 1 per week , 2-4 per week , 5-6 per week , 1 per day , 2-3 per day , 4-5 per day , 6+ per day |
| q17_apricot | Apricots (1 fresh, ½ cup canned or 5 dried) | Never, or less than once per month , 1-3 per month , 1 per week , 2-4 per week , 5-6 per week , 1 per day , 2-3 per day , 4-5 per day , 6+ per day |

**Physical Activity Survey**

| **Variable Name** | **Corresponding Questions** | **Question Options** |
| --- | --- | --- |
| Starting Prompt: N/A | | Type: Radio Button |
| q1_time_walk_exerc | How much time in the past week did you spend walking for exercise? Do not include time spent walking for transportation. | Zero , 1-4 min , 5-19 min , 20-59 min , One hour , 1-1.5 hrs , 2-3 hrs , 4-6 hrs , 7-10 hrs , 11+ hrs |
| q2_time_mod_int_exerc | How much time in the past week did you spend doing moderate-intensity exercise? In general, during moderate-intensity exercise, you can talk but not sing. | Zero , 1-4 min , 5-19 min , 20-59 min , One hour , 1-1.5 hrs , 2-3 hrs , 4-6 hrs , 7-10 hrs , 11+ hrs |
| q3_time_vig_int_exerc | How much time in the past week did you spend doing vigorous-intensity exercise? In general, during vigorous-intensity exercise, you cannot say more than a few words without pausing for a breath. | Zero , 1-4 min , 5-19 min , 20-59 min , One hour , 1-1.5 hrs , 2-3 hrs , 4-6 hrs , 7-10 hrs , 11+ hrs |
| q4_weight_resis_exerc | How much time in the past week did you spend weight training or doing resistance exercises? | Zero , 1-4 min , 5-19 min , 20-59 min , One hour , 1-1.5 hrs , 2-3 hrs , 4-6 hrs , 7-10 hrs , 11+ hrs |

**Nuts and Dairy Survey**

| **Variable Name** | **Corresponding Questions** | **Question Options** |
| --- | --- | --- |
| Starting Prompt: Please indicate how often you consumed the specified amount of each food during the past 6 months. | | Type: Radio Button |
| q1_skim_milk | Skim milk (8 oz.) | Never, or less than once per month , 1-3 per month , 1 per week , 2-4 per week , 5-6 per week , 1 per day , 2-3 per day , 4-5 per day , 6+ per day |
| q2_low_fat_milk | 1% or 2% milk (8 oz.) | Never, or less than once per month , 1-3 per month , 1 per week , 2-4 per week , 5-6 per week , 1 per day , 2-3 per day , 4-5 per day , 6+ per day |
| q3_whole_milk | Whole milk (8 oz.) | Never, or less than once per month , 1-3 per month , 1 per week , 2-4 per week , 5-6 per week , 1 per day , 2-3 per day , 4-5 per day , 6+ per day |
| q4_soy_milk | Soy milk (8 oz.) | Never, or less than once per month , 1-3 per month , 1 per week , 2-4 per week , 5-6 per week , 1 per day , 2-3 per day , 4-5 per day , 6+ per day |
| q5_cream | Cream, e.g., coffee cream, whipped or sour cream (1 tbsp.) | Never, or less than once per month , 1-3 per month , 1 per week , 2-4 per week , 5-6 per week , 1 per day , 2-3 per day , 4-5 per day , 6+ per day |
| q6_non_mil_caff | Non-dairy coffee whitener (1 tbsp.) | Never, or less than once per month , 1-3 per month , 1 per week , 2-4 per week , 5-6 per week , 1 per day , 2-3 per day , 4-5 per day , 6+ per day |
| q7_ice_cream | Frozen yogurt, sherbet, or low-fat ice cream (1 cup) | Never, or less than once per month , 1-3 per month , 1 per week , 2-4 per week , 5-6 per week , 1 per day , 2-3 per day , 4-5 per day , 6+ per day |
| q8_low_carb_yogurt | Low-carb yogurt, artificially sweetened or plain (1 cup) | Never, or less than once per month , 1-3 per month , 1 per week , 2-4 per week , 5-6 per week , 1 per day , 2-3 per day , 4-5 per day , 6+ per day |
| q9_fruit_yogurt | Yogurt sweetened with fruit or other flavoring (1 cup) | Never, or less than once per month , 1-3 per month , 1 per week , 2-4 per week , 5-6 per week , 1 per day , 2-3 per day , 4-5 per day , 6+ per day |
| q10_marg_spread | Margarine as a spread (exclude use in cooking) | Never, or less than once per month , 1-3 per month , 1 per week , 2-4 per week , 5-6 per week , 1 per day , 2-3 per day , 4-5 per day , 6+ per day |
| q11_butter_spread | Pure butter as a spread (exclude use in cooking) | Never, or less than once per month , 1-3 per month , 1 per week , 2-4 per week , 5-6 per week , 1 per day , 2-3 per day , 4-5 per day , 6+ per day |
| q12_cott_rico_cheese | Cottage or ricotta cheese (½ cup) | Never, or less than once per month , 1-3 per month , 1 per week , 2-4 per week , 5-6 per week , 1 per day , 2-3 per day , 4-5 per day , 6+ per day |
| q13_cream_cheese | Cream cheese (1 oz.) | Never, or less than once per month , 1-3 per month , 1 per week , 2-4 per week , 5-6 per week , 1 per day , 2-3 per day , 4-5 per day , 6+ per day |
| q14_other_cheese | Other cheese, e.g., American, cheddar, plain or as a part of a dish (1 slice or 1 oz. serving) | Never, or less than once per month , 1-3 per month , 1 per week , 2-4 per week , 5-6 per week , 1 per day , 2-3 per day , 4-5 per day , 6+ per day |
| q15_cheese_type | What type of cheese do you usually eat? | Never, or less than once per month , 1-3 per month , 1 per week , 2-4 per week , 5-6 per week , 1 per day , 2-3 per day , 4-5 per day , 6+ per day |
| q16_peanut | Peanuts (small packet or 1 oz.) | Never, or less than once per month , 1-3 per month , 1 per week , 2-4 per week , 5-6 per week , 1 per day , 2-3 per day , 4-5 per day , 6+ per day |
| q17_walnut | Walnuts (1 oz.) | Never, or less than once per month , 1-3 per month , 1 per week , 2-4 per week , 5-6 per week , 1 per day , 2-3 per day , 4-5 per day , 6+ per day |
| q18_other_nut | Other nuts (small packet or 1 oz.) | Never, or less than once per month , 1-3 per month , 1 per week , 2-4 per week , 5-6 per week , 1 per day , 2-3 per day , 4-5 per day , 6+ per day |

**Beverages Survey**

| **Variable Name** | **Corresponding Questions** | **Question Options** |
| --- | --- | --- |
| Starting Prompt: Please indicate how often you consumed the specified amount of each beverage during the past 6 months. | | Type: Radio Button |
| q1_low_cal_wt_caf | Low-calorie beverage with caffeine, e.g., Diet Coke, Diet Mtn. Dew (1 glass, bottle, can) | Never, or less than once per month , 1-3 per month , 1 per week , 2-4 per week , 5-6 per week , 1 per day , 2-3 per day , 4-5 per day , 6+ per day |
| q2_low_cal_wo_caf | Other low-calorie beverage without caffeine, e.g., Diet 7-Up (1 glass, bottle, can) | Never, or less than once per month , 1-3 per month , 1 per week , 2-4 per week , 5-6 per week , 1 per day , 2-3 per day , 4-5 per day , 6+ per day |
| q3_carb_bever_wt_sugar | Other carbonated beverage with sugar, e.g., 7-Up, root beer, ginger ale, Caffeine-Free Coke (1 glass, bottle can) | Never, or less than once per month , 1-3 per month , 1 per week , 2-4 per week , 5-6 per week , 1 per day , 2-3 per day , 4-5 per day , 6+ per day |
| q4_sugar_bever | Other sugared beverages: punch, lemonade, sports drinks, or sugared ice tea (1 glass, bottle, can) | Never, or less than once per month , 1-3 per month , 1 per week , 2-4 per week , 5-6 per week , 1 per day , 2-3 per day , 4-5 per day , 6+ per day |
| q5_reg_beer | Beer, regular (1 glass, bottle, can) | Never, or less than once per month , 1-3 per month , 1 per week , 2-4 per week , 5-6 per week , 1 per day , 2-3 per day , 4-5 per day , 6+ per day |
| q6_light_beer | Light beer, e.g., Bud Light (1 glass, bottle, can) | Never, or less than once per month , 1-3 per month , 1 per week , 2-4 per week , 5-6 per week , 1 per day , 2-3 per day , 4-5 per day , 6+ per day |
| q7_red_wine | Red wine (5 oz. glass) | Never, or less than once per month , 1-3 per month , 1 per week , 2-4 per week , 5-6 per week , 1 per day , 2-3 per day , 4-5 per day , 6+ per day |
| q8_white_wine | White wine (5 oz. glass) | Never, or less than once per month , 1-3 per month , 1 per week , 2-4 per week , 5-6 per week , 1 per day , 2-3 per day , 4-5 per day , 6+ per day |
| q9_liquor | Liquor, e.g., vodka, gin (1 drink or shot) | Never, or less than once per month , 1-3 per month , 1 per week , 2-4 per week , 5-6 per week , 1 per day , 2-3 per day , 4-5 per day , 6+ per day |
| q10_water | Water: bottled, sparkling, or tap (8 oz. cup) | Never, or less than once per month , 1-3 per month , 1 per week , 2-4 per week , 5-6 per week , 1 per day , 2-3 per day , 4-5 per day , 6+ per day |
| q11_decaf_tea | Herbal tea or decaffeinated tea ( 8 oz. cup) | Never, or less than once per month , 1-3 per month , 1 per week , 2-4 per week , 5-6 per week , 1 per day , 2-3 per day , 4-5 per day , 6+ per day |
| q12_caf_tea | Tea with caffeine (8 oz. cup), including green tea | Never, or less than once per month , 1-3 per month , 1 per week , 2-4 per week , 5-6 per week , 1 per day , 2-3 per day , 4-5 per day , 6+ per day |
| q13_decaf_coffee | Decaffeinated coffee (8 oz. cup) | Never, or less than once per month , 1-3 per month , 1 per week , 2-4 per week , 5-6 per week , 1 per day , 2-3 per day , 4-5 per day , 6+ per day |
| q14_caf_coffee | Coffee with caffeine (8 oz. cup) | Never, or less than once per month , 1-3 per month , 1 per week , 2-4 per week , 5-6 per week , 1 per day , 2-3 per day , 4-5 per day , 6+ per day |
| q15_dairy_coff_drink | Dairy coffee drink (hot/cold) e.g., cappuccino (16 oz.) | Never, or less than once per month , 1-3 per month , 1 per week , 2-4 per week , 5-6 per week , 1 per day , 2-3 per day , 4-5 per day , 6+ per day |

**Sleep Survey**

| **Variable Name** | **Corresponding Questions** | **Question Options** |
| --- | --- | --- |
| Starting Prompt: N/A | | Type: Radio Button |
| q1_time_sleep | Last time you slept, what time did you fall asleep? Please round to the nearest hour. | 12:00 midnight , 1:00 am , 2:00 am , 3:00 am , 4:00 am , 5:00 am , 6:00 am , 7:00 am , 8:00 am , 9:00 am , 10:00 am , 11:00 am , 12:00 noon , 1:00 pm , 2:00 pm , 3:00 pm , 4:00 pm , 5:00 pm , 6:00 pm , 7:00 pm , 8:00 pm , 9:00 pm , 10:00 pm , 11:00 pm |
| q2_time_wake_up | What time did you wake up? | 12:00 midnight , 1:00 am , 2:00 am , 3:00 am , 4:00 am , 5:00 am , 6:00 am , 7:00 am , 8:00 am , 9:00 am , 10:00 am , 11:00 am , 12:00 noon , 1:00 pm , 2:00 pm , 3:00 pm , 4:00 pm , 5:00 pm , 6:00 pm , 7:00 pm , 8:00 pm , 9:00 pm , 10:00 pm , 11:00 pm |
| q3_sleep_qual | Please rate the quality of your sleep. | Very bad , Fairly bad , Fairly good , Very good |

**Meats Survey**

| **Variable Name** | **Corresponding Questions** | **Question Options** |
| --- | --- | --- |
| Starting Prompt: Please indicate how often you consumed the specified amount of each food during the past 6 months. | | Type: Radio Button |
| q1_omega3_egg | Omega-3 fortified eggs including yolk | Never, or less than once per month , 1-3 per month , 1 per week , 2-4 per week , 5-6 per week , 1 per day , 2-3 per day , 4-5 per day , 6+ per day |
| q2_regu_egg | Regular eggs including yolk | Never, or less than once per month , 1-3 per month , 1 per week , 2-4 per week , 5-6 per week , 1 per day , 2-3 per day , 4-5 per day , 6+ per day |
| q3_beef_pork_hotd | Beef or pork hot dogs (1) | Never, or less than once per month , 1-3 per month , 1 per week , 2-4 per week , 5-6 per week , 1 per day , 2-3 per day , 4-5 per day , 6+ per day |
| q4_chic_turk_hotd | Chicken or turkey hot dogs or sausage (1) | Never, or less than once per month , 1-3 per month , 1 per week , 2-4 per week , 5-6 per week , 1 per day , 2-3 per day , 4-5 per day , 6+ per day |
| q5_chic_turk_sandw | Chicken/turkey sandwich or frozen dinner | Never, or less than once per month , 1-3 per month , 1 per week , 2-4 per week , 5-6 per week , 1 per day , 2-3 per day , 4-5 per day , 6+ per day |
| q6_chic_turk_other_wt_skin | Other chicken or turkey, with skin (3 oz.) | Never, or less than once per month , 1-3 per month , 1 per week , 2-4 per week , 5-6 per week , 1 per day , 2-3 per day , 4-5 per day , 6+ per day |
| q7_chic_turk_other_wo_skin | Other chicken or turkey, without skin (including ground turkey) (3 oz.) | Never, or less than once per month , 1-3 per month , 1 per week , 2-4 per week , 5-6 per week , 1 per day , 2-3 per day , 4-5 per day , 6+ per day |
| q8_bacon | Bacon (2 slices) | Never, or less than once per month , 1-3 per month , 1 per week , 2-4 per week , 5-6 per week , 1 per day , 2-3 per day , 4-5 per day , 6+ per day |
| q9_meat_sandw_other | Salami, bologna, or other processed meat sandwiches | Never, or less than once per month , 1-3 per month , 1 per week , 2-4 per week , 5-6 per week , 1 per day , 2-3 per day , 4-5 per day , 6+ per day |
| q10_proc_meat_other | Other processed meats, e.g., sausage, kielbasa (2 oz. or 2 small links) | Never, or less than once per month , 1-3 per month , 1 per week , 2-4 per week , 5-6 per week , 1 per day , 2-3 per day , 4-5 per day , 6+ per day |
| q11_lean_hamb | Lean or extra lean hamburger (1 patty) | Never, or less than once per month , 1-3 per month , 1 per week , 2-4 per week , 5-6 per week , 1 per day , 2-3 per day , 4-5 per day , 6+ per day |
| q12_reg_hamb | Regular hamburger (1 patty) | Never, or less than once per month , 1-3 per month , 1 per week , 2-4 per week , 5-6 per week , 1 per day , 2-3 per day , 4-5 per day , 6+ per day |
| q13_sand_mix_dish | Beef, pork, or lab as a sandwich or mixed dish, e.g., stew, casserole, lasagna, frozen dinners | Never, or less than once per month , 1-3 per month , 1 per week , 2-4 per week , 5-6 per week , 1 per day , 2-3 per day , 4-5 per day , 6+ per day |
| q14_pork_main_dish | Pork as a main dish, e.g., ham or chops (4-6 oz.) | Never, or less than once per month , 1-3 per month , 1 per week , 2-4 per week , 5-6 per week , 1 per day , 2-3 per day , 4-5 per day , 6+ per day |
| q15_beef_lamb_main_dish | Beef or lamb as a main dish, e.g., steak, roast (4-6 oz.) | Never, or less than once per month , 1-3 per month , 1 per week , 2-4 per week , 5-6 per week , 1 per day , 2-3 per day , 4-5 per day , 6+ per day |
| q16_can_tuna | Canned tuna fish (3-4 oz.) | Never, or less than once per month , 1-3 per month , 1 per week , 2-4 per week , 5-6 per week , 1 per day , 2-3 per day , 4-5 per day , 6+ per day |
| q17_bread_fish | Breaded fish cakes, pieces, or fish sticks (1 serving, store bought) | Never, or less than once per month , 1-3 per month , 1 per week , 2-4 per week , 5-6 per week , 1 per day , 2-3 per day , 4-5 per day , 6+ per day |
| q18_shell_fish | Shrimp, lobster, scallops as a main dish | Never, or less than once per month , 1-3 per month , 1 per week , 2-4 per week , 5-6 per week , 1 per day , 2-3 per day , 4-5 per day , 6+ per day |
| q19_dark_meat_fish | Dark meat fish, e.g., tuna steak, mackerel, salmon, sardines, bluefish, swordfish (3-5 oz.) | Never, or less than once per month , 1-3 per month , 1 per week , 2-4 per week , 5-6 per week , 1 per day , 2-3 per day , 4-5 per day , 6+ per day |
| q20_other_fish | Other fish, e.g., cod, haddock, halibut (3-5 oz.) | Never, or less than once per month , 1-3 per month , 1 per week , 2-4 per week , 5-6 per week , 1 per day , 2-3 per day , 4-5 per day , 6+ per day |

**Pets Survey**

| **Variable Name** | **Corresponding Questions** | **Question Options** |
| --- | --- | --- |
| Starting Prompt: N/A | | Type: Radio Button |
| q1_dog_in_household | Are there one or more dogs in your household? | Yes, No |
| q2_num_of_dog* | How many dogs? | One , Two , Three , Four , Five or more |
| q3_breed_of_dog* | Which breed(s)? | (Free Response) Single-line Text |
| q4_cat_in_household | Are there one or more cats in your household? | Yes, No |
| q5_num_of_cat* | How many cats? | One , Two , Three , Four , Five or more |
| q6_breed_of_cat* | Which breed(s)? | (Free Response) Single-line Text |
| ** Conditionally displayed* | | |

**Stress/Enjoy Survey**

| **Variable Name** | **Corresponding Questions** | **Question Options** |
| --- | --- | --- |
| Starting Prompt: N/A | | Type: Radio Button |
| q1_stress_past_hr | Did anything stressful happen in the past hour? | Yes, No |
| q2_stress_lvl* | How stressful was this for you? | A little , Somewhat , Very |
| q3_enjoy_past_hr | Did anything enjoyable happen in the past hour? | Yes, No |
| Q4_enjoy_lvl* | How enjoyable was this for you? | A little , Somewhat , Very |
| ** Conditionally displayed* | | |

**Sitting Survey**

| **Variable Name** | **Corresponding Questions** | **Question Options** |
| --- | --- | --- |
| Starting Prompt: N/A | | Type: Radio Button |
| q1_tot_hr_sit | In the past 24 hours, what was the total time you spent sitting? (Include transportation, at work, leisure time, meal times) | Less than 1 hour , 1 hour , 2 hours , 3 hours , 4 hours , 5 hours , 6 hours , 7 hours , 8 hours , 9 hours , 10 hours , 11 hours , 12+ hours |

**Emotions Survey**

| **Variable Name** | **Corresponding Questions** | **Question Options** |
| --- | --- | --- |
| Starting Prompt: N/A | | Type: Check |
| q1_feel | Right now I am: | Content , Interested , Determined , Optimistic , Confident , Happy , Valued , Excited , Stressed , Confused , Frustrated , Anxious , Insecure , Angry , Lonely , Tired |

**Vegetables Survey**

| **Variable Name** | **Corresponding Questions** | **Question Options** |
| --- | --- | --- |
| Starting Prompt: Please indicate how often you consumed the specified amount of each food during the past 4 months. | | Type: Radio Button |
| q1_tomato_2slice | Tomatoes (2 slices) | Never, or less than once per month , 1-3 per month , 1 per week , 2-4 per week , 5-6 per week , 1 per day , 2-3 per day , 4-5 per day , 6+ per day |
| q2_tomato_or_juice | Tomato or V-8 juice (small glass) | Never, or less than once per month , 1-3 per month , 1 per week , 2-4 per week , 5-6 per week , 1 per day , 2-3 per day , 4-5 per day , 6+ per day |
| q3_tomato_sauce | Tomato sauce (½ cup) e.g., spaghetti sauce | Never, or less than once per month , 1-3 per month , 1 per week , 2-4 per week , 5-6 per week , 1 per day , 2-3 per day , 4-5 per day , 6+ per day |
| q4_salsa_pican_taco_sauce | Salsa, picante or taco sauce (¼ cup) | Never, or less than once per month , 1-3 per month , 1 per week , 2-4 per week , 5-6 per week , 1 per day , 2-3 per day , 4-5 per day , 6+ per day |
| q5_string_bean | String beans (½ cup) | Never, or less than once per month , 1-3 per month , 1 per week , 2-4 per week , 5-6 per week , 1 per day , 2-3 per day , 4-5 per day , 6+ per day |
| q6_bean_or_lentil | Beans or lentils, baked, dried, or soup (½ cup) | Never, or less than once per month , 1-3 per month , 1 per week , 2-4 per week , 5-6 per week , 1 per day , 2-3 per day , 4-5 per day , 6+ per day |
| q7_other_soy_protein | Tofu, soy burger, soybeans, miso, or other soy protein | Never, or less than once per month , 1-3 per month , 1 per week , 2-4 per week , 5-6 per week , 1 per day , 2-3 per day , 4-5 per day , 6+ per day |
| q8_pea_or_lima_bean | Peas or lima beans (½ cup fresh, frozen, canned) | Never, or less than once per month , 1-3 per month , 1 per week , 2-4 per week , 5-6 per week , 1 per day , 2-3 per day , 4-5 per day , 6+ per day |
| q9_broccoli | Broccoli (½ cup) | Never, or less than once per month , 1-3 per month , 1 per week , 2-4 per week , 5-6 per week , 1 per day , 2-3 per day , 4-5 per day , 6+ per day |
| q10_cauliflower | Cauliflower (½ cup | Never, or less than once per month , 1-3 per month , 1 per week , 2-4 per week , 5-6 per week , 1 per day , 2-3 per day , 4-5 per day , 6+ per day |
| q11_cabbage_or_coleslaw | Cabbage or coleslaw (½ cup) | Never, or less than once per month , 1-3 per month , 1 per week , 2-4 per week , 5-6 per week , 1 per day , 2-3 per day , 4-5 per day , 6+ per day |
| q12_brussel_sprout | Brussels sprouts (½ cup) | Never, or less than once per month , 1-3 per month , 1 per week , 2-4 per week , 5-6 per week , 1 per day , 2-3 per day , 4-5 per day , 6+ per day |
| q13_raw_carrot | Carrots, raw (½ carrot or 2-4 sticks) | Never, or less than once per month , 1-3 per month , 1 per week , 2-4 per week , 5-6 per week , 1 per day , 2-3 per day , 4-5 per day , 6+ per day |
| q14_cooked_carrot | Carrots, cooked (½ cup) or carrot juice (2-3 oz.) | Never, or less than once per month , 1-3 per month , 1 per week , 2-4 per week , 5-6 per week , 1 per day , 2-3 per day , 4-5 per day , 6+ per day |
| q15_corn | Corn (1 ear or ½ cup frozen or canned) | Never, or less than once per month , 1-3 per month , 1 per week , 2-4 per week , 5-6 per week , 1 per day , 2-3 per day , 4-5 per day , 6+ per day |
| q16_mixed_or_stirfry_veg | Mixed or stir-fry vegetables (½ cup) or vegetable soup (1 cup) | Never, or less than once per month , 1-3 per month , 1 per week , 2-4 per week , 5-6 per week , 1 per day , 2-3 per day , 4-5 per day , 6+ per day |
| q17_yam_or_sweet_potato | Yams or sweet potatoes (½ cup) | Never, or less than once per month , 1-3 per month , 1 per week , 2-4 per week , 5-6 per week , 1 per day , 2-3 per day , 4-5 per day , 6+ per day |
| q18_winter_squash | Dark orange (winter) squash (½ cup) | Never, or less than once per month , 1-3 per month , 1 per week , 2-4 per week , 5-6 per week , 1 per day , 2-3 per day , 4-5 per day , 6+ per day |
| q19_summer_squash | Eggplant, zucchini, or other summer squash (½ cup) | Never, or less than once per month , 1-3 per month , 1 per week , 2-4 per week , 5-6 per week , 1 per day , 2-3 per day , 4-5 per day , 6+ per day |
| q20_kale_mustard_green_chard | Kale, mustard greens, or chard (½ cup) | Never, or less than once per month , 1-3 per month , 1 per week , 2-4 per week , 5-6 per week , 1 per day , 2-3 per day , 4-5 per day , 6+ per day |
| q21_cooked_spinach | Spinach, cooked (½ cup) | Never, or less than once per month , 1-3 per month , 1 per week , 2-4 per week , 5-6 per week , 1 per day , 2-3 per day , 4-5 per day , 6+ per day |
| q22_raw_spinach | Spinach, raw as in salad (1 cup) | Never, or less than once per month , 1-3 per month , 1 per week , 2-4 per week , 5-6 per week , 1 per day , 2-3 per day , 4-5 per day , 6+ per day |
| q23_iceberg_or_head_lettuce | Iceberg or head lettuce (1 serving) | Never, or less than once per month , 1-3 per month , 1 per week , 2-4 per week , 5-6 per week , 1 per day , 2-3 per day , 4-5 per day , 6+ per day |
| q24_romaine_or_leaf_lettuce | Romaine or leaf lettuce (1 serving) | Never, or less than once per month , 1-3 per month , 1 per week , 2-4 per week , 5-6 per week , 1 per day , 2-3 per day , 4-5 per day , 6+ per day |
| q25_celery | Celery (2-3 sticks) | Never, or less than once per month , 1-3 per month , 1 per week , 2-4 per week , 5-6 per week , 1 per day , 2-3 per day , 4-5 per day , 6+ per day |
| q26_peppers_tri_color | Peppers: green, yellow, or red (3 slices) | Never, or less than once per month , 1-3 per month , 1 per week , 2-4 per week , 5-6 per week , 1 per day , 2-3 per day , 4-5 per day , 6+ per day |
| q27_raw_onion | Onions as a garnish or in salad (1 slice) | Never, or less than once per month , 1-3 per month , 1 per week , 2-4 per week , 5-6 per week , 1 per day , 2-3 per day , 4-5 per day , 6+ per day |
| q28_cooked_onion | Onions as a cooked vegetable, rings, or soup (½ cup) | Never, or less than once per month , 1-3 per month , 1 per week , 2-4 per week , 5-6 per week , 1 per day , 2-3 per day , 4-5 per day , 6+ per day |
