# Supplementary material for: Measuring Environmental and Behavioral Drivers of Chronic Diseases Using Smartphone-Based Digital Phenotyping: Intensive Longitudinal Observational mHealth Substudy Embedded in 2 Prospective Cohorts of Adults
Source: JMIR Public Health Surveill. 2024 Oct 11;10:e55170. doi: 10.2196/55170 (PMC11512133; doi:10.2196/55170)
Supplement: Multimedia Appendix 4 [file publichealth_v10i1e55170_app4.docx]

**Table S2.** Demographic characteristics of active participants in Nurses’ Health Study 3 (NHS3) and Growing Up Today Study (GUTS) cohorts at baseline who were invited or not invited to the study, and invited participants who consented or did not consent.

| Variables | Active cohort at the beginning of Substudy who were invited  n = 32,430 | Active cohort at the beginning of Substudy who were not invited  n = 13,691 | |
| --- | --- | --- | --- |
| **Age (years), mean (SD)** | 41.4 (7.8) | 39.2 (7.7) |  |
| **Sex, n (%)** |  |  |  |
| Male | 1,590 (4.9%) | 2,337 (17%) |  |
| Female | 30,840 (95%) | 11,354 (83%) |  |
| **Race, n (%)** |  |  |  |
| White | 29,718 (92%) | 12,327 (90%) |  |
| Black or African American | 851 (2.6%) | 379 (2.8%) |  |
| American Indian or Alaska Native | 369 (1.1%) | 130 (0.9%) |  |
| Asian | 906 (2.8%) | 503 (3.7%) |  |
| Native Hawaiian or Other Pacific Islander | 118 (0.4%) | 70 (0.5%) |  |
| **Ethnicity, n (%)** |  |  |  |
| Hispanic or Latino | 1,313 (4.0%) | 538 (3.9%) |  |
| Not Hispanic or Latino | 30,985 (96%) | 13,068 (95%) |  |
| **Married, n (%)** | 18,971 (59%) | 8,269 (61%) |  |
| **Smoking Status, n (%)** |  |  |  |
| Never | 24,392 (75%) | 10,222 (75%) |  |
| Current | 1,487 (4.6%) | 651 (4.8%) |  |
| Former | 6,364 (20%) | 2,721 (20%) |  |
| **Body Mass Index (kg/m²), mean (SD)** | 26.7 (6.4) | 26.7 (6.2) |  |
| **Annual household income** |  |  |  |
| Less than $30,000 | 395 (2.9%) | 745 (2.3%) |  |
| $30,000 to $50,000 | 530 (3.9%) | 1,074 (3.3%) |  |
| $50,000 to $70,000 | 916 (6.7%) | 2,327 (7.2%) |  |
| $70,000 to $90,000 | 1,123 (8.2%) | 2,873 (8.9%) |  |
| $90,000 to $200,000 | 3,675 (27%) | 10,934 (34%) |  |
| More than $200,000 | 908 (6.6%) | 2,590 (8.0%) |  |
| Missing/Not Provided | 6,144 (45%) | 11,887 (37%) |  |
