# Supplementary material for: Measuring Environmental and Behavioral Drivers of Chronic Diseases Using Smartphone-Based Digital Phenotyping: Intensive Longitudinal Observational mHealth Substudy Embedded in 2 Prospective Cohorts of Adults
Source: JMIR Public Health Surveill. 2024 Oct 11;10:e55170. doi: 10.2196/55170 (PMC11512133; doi:10.2196/55170)
Supplement: Multimedia Appendix 5 [file publichealth_v10i1e55170_app5.docx]

**Table S3.** Demographic characteristics of participants in the Beiwe Smartphone Substudy of Nurses' Health Study 3 (NHS3) and Growing Up Today Study (GUTS) provided at least 30, 60, and 90 days of data

|  | **Provided at least 30 days of data** | **Provided at least 60 days of data** | **Provided at least 90 days of data** |
| --- | --- | --- | --- |
| **Variable** | **N = 1,867** | **N = 1,669** | **N = 1,371** |
| **Age (years), Mean (SD)** | 41.7 (8.1) | 41.7 (8.1) | 41.8 (8.0) |
| **Sex, n (%)** |  |  |  |
| Male | 120 (6.4%) | 111 (6.7%) | 87 (6.3%) |
| Female | 1,747 (94%) | 1,558 (93%) | 1,284 (94%) |
| **Race, n (%)** |  |  |  |
| White | 1,746 (94%) | 1,560 (93%) | 1,279 (93%) |
| Black or African American | 41 (2.2%) | 37 (2.2%) | 33 (2.4%) |
| American Indian or Alaska Native | 19 (1.0%) | 18 (1.1%) | 15 (1.1%) |
| Asian | 36 (1.9%) | 34 (2.0%) | 26 (1.9%) |
| Native Hawaiian or Other Pacific Islander | 5 (0.3%) | 2 (0.1%) | 2 (0.1%) |
| **Ethnicity, n (%)** |  |  |  |
| Hispanic or Latino | 63 (3.4%) | 56 (3.4%) | 42 (3.1%) |
| Not Hispanic or Latino | 1,802 (97%) | 1,611 (97%) | 1,327 (97%) |
| **Married, n (%)** | 1,112 (60%) | 988 (60%) | 821 (60%) |
| **Smoking Status, n (%)** |  |  |  |
| Never | 1,431 (77%) | 1,279 (77%) | 1,056 (77%) |
| Current | 74 (4.0%) | 68 (4.1%) | 58 (4.2%) |
| Former | 356 (19%) | 316 (19%) | 252 (18%) |
|  |  |  |  |
| **Body Mass Index (kg/m²), Mean (SD)** | 27.3 (6.7) | 27.3 (6.7) | 27.4 (6.7) |
| **Annual Household Income, n (%)** |  |  |  |
| Less than $30,000 | 74 (4.0%) | 69 (4.1%) | 56 (4.1%) |
| $30,000 to $50,000 | 90 (4.8%) | 79 (4.7%) | 65 (4.7%) |
| $50,000 to $70,000 | 193 (10%) | 173 (10%) | 134 (9.8%) |
| $70,000 to $90,000 | 249 (13%) | 228 (14%) | 191 (14%) |
| $90,000 to $200,000 | 873 (47%) | 776 (46%) | 648 (47%) |
| More than $200,000 | 226 (12%) | 208 (12%) | 170 (12%) |
| Missing/Not Provided | 162 (8.7%) | 136 (8.1%) | 107 (7.8%) |
| **Phone Operating System (OS), n (%)** |  |  |  |
| Android | 472 (25%) | 405 (24%) | 324 (24%) |
| iOS | 1,390 (74%) | 1,259 (75%) | 1,042 (76%) |
| Both^a^ | 5 (0.3%) | 5 (0.3%) | 5 (0.4%) |

*Notes.*

^a^ Some participants switched smartphones during the one-year data collection period, which resulted in a different OS.
