# Supplementary material for: Measuring Environmental and Behavioral Drivers of Chronic Diseases Using Smartphone-Based Digital Phenotyping: Intensive Longitudinal Observational mHealth Substudy Embedded in 2 Prospective Cohorts of Adults
Source: JMIR Public Health Surveill. 2024 Oct 11;10:e55170. doi: 10.2196/55170 (PMC11512133; doi:10.2196/55170)
Supplement: Multimedia Appendix 6 [file publichealth_v10i1e55170_app6.docx]

| **Table S4.** GPS and accelerometer data compliance for the Beiwe Smartphone Substudy of Nurses' Health Study 3 (NHS3) and Growing Up Today Study (GUTS). |
| --- |

|  | ***GPS data*** | | | | | | |
| --- | --- | --- | --- | --- | --- | --- | --- |
|  | **Overall** | **By phone OS** | | | |  |  |
|  | **n = 443,790** | **Android, n = 100,300** | **iOS, n = 341,884** | **Both^a^, n = 1,6**06 | |  |  |
| **Valid hours** |  |  |  | |  |  |  |
| Mean (SD) | 14.8 (5.9) | 19.8 (6.1) | 13.3 (5.0) | 15.4 (6.3) | |  |  |
| Median (IQR) | 14.3 (10.8, 19.0) | 23.3 (17.5, 24.0) | 13.3 (10.0, 16.3) | 15.3 (10.5, 21.8) | |  |  |

|  | ***Accelerometer data*** | | | | | | |
| --- | --- | --- | --- | --- | --- | --- | --- |
|  | **Overall** | **By phone OS** | | | |  |  |
|  | **n = 461,484** | **Android, n = 116,327** | **iOS, n = 343,499** | | **Both^a^, n = 1,658** |  |  |
| **Valid hours** |  |  |  |  | |  |  |
| Mean (SD) | 13.2 (4.8) | 13.8 (4.7) | 13.0 (4.8) | | 12.2 (5.4) |  |  |
| Median (IQR) | 13.2 (10.1, 16.2) | 14.0 (10.7, 17.0) | 12.9 (9.9, 16.0) | | 12.1 (8.5, 15.6) |  |  |

^a^ Some participants switched smartphones during the one-year data collection period, which resulted in a different OS.
