# Supplementary material for: Measuring Environmental and Behavioral Drivers of Chronic Diseases Using Smartphone-Based Digital Phenotyping: Intensive Longitudinal Observational mHealth Substudy Embedded in 2 Prospective Cohorts of Adults
Source: JMIR Public Health Surveill. 2024 Oct 11;10:e55170. doi: 10.2196/55170 (PMC11512133; doi:10.2196/55170)
Supplement: Multimedia Appendix 7 [file publichealth_v10i1e55170_app7.docx]

| **Table S5.** Smartphone GPS and accelerometer data compliance at participant-day level based on participant follow-up period by phone operating system (OS) for the Beiwe Smartphone Substudy of Nurses’ Health Study 3 (NHS3) and Growing Up Today Study (GUTS). |
| --- |

|  | **Overall** | **By phone OS** | | |
| --- | --- | --- | --- | --- |
|  | **N = 511,161** | **Android, N = 121,077** | **iOS, N = 388,235** | **Both^a^, N = 1,676** |
|  | | | | |
| **Participant-day compliance rates - GPS data** | | | | |
| Valid days | 351,520 (69%) | 90,410 (75%) | 259,838 (67%) | 1,272 (69%) |
| Invalid days | 92,270 (18%) | 9,890 (8.2%) | 82,046 (21%) | 334 (18%) |
| Missing days^b^ | 67,371 (13%) | 20,777 (17%) | 46,351 (12%) | 243 (13%) |
|  | | | | |
| **Participant-day compliance rates - accelerometer data** | | | | |
| Valid days | 348,394 (68%) | 91,960 (76%) | 255,333 (66%) | 1,101 (60%) |
| Invalid days | 113,090 (22%) | 24,367 (20%) | 88,166 (23%) | 557 (30%) |
| Missing days^b^ | 49,677 (9.7%) | 4,750 (3.9%) | 44,736 (12%) | 191 (10%) |

| ^a^Some participants switched smartphones during the one-year data collection period, which resulted in a different OS.  ^b^Missing days were defined as days in the follow-up period when data were missing owing to sensor non-collection. Reasons could include a participant forgetting to charge their phone, disabling GPS, or a major update from the OS causing the Beiwe app to malfunction. |
| --- |
