# Supplementary material for: Measuring Environmental and Behavioral Drivers of Chronic Diseases Using Smartphone-Based Digital Phenotyping: Intensive Longitudinal Observational mHealth Substudy Embedded in 2 Prospective Cohorts of Adults
Source: JMIR Public Health Surveill. 2024 Oct 11;10:e55170. doi: 10.2196/55170 (PMC11512133; doi:10.2196/55170)
Supplement: Multimedia Appendix 8 [file publichealth_v10i1e55170_app8.docx]

| **Table S6.** The participant-day level smartphone-based GPS and accelerometer data compliance by two validity criteria. |
| --- |

|  | **≥10 valid hours** | **≥20 valid hours** |
| --- | --- | --- |
|  | **N = 870,525** | |
| **Participant-day compliance rates - GPS data** | | |
| Valid days | 351,520 (40%) | 99,694 (11%) |
| Invalid days | 92,270 (11%) | 344,096 (40%) |
| Non-collection days^a^ | 426,735 (49%) | 426,735 (49%) |
|  |  |  |
| **Participant-day compliance rates - accelerometer data** | | |
| Valid days | 348,394 (40%) | 38,613 (4%) |
| Invalid days | 113,090 (13%) | 422,871 (49%) |
| Non-collection days^a^ | 409,041 (47%) | 409,041 (47%) |

^a^ Non-collection days were defined as days when data were missing owing to sensor non-collection. In this study, the main reason for the non-collection days was that participants uninstalled the Beiwe app before the completion date of their one-year study. Other reasons could include a participant forgetting to charge their phone, disabling GPS, or a major update from the OS causing the Beiwe app to malfunction temporarily.
