# Supplementary material for: Measuring Environmental and Behavioral Drivers of Chronic Diseases Using Smartphone-Based Digital Phenotyping: Intensive Longitudinal Observational mHealth Substudy Embedded in 2 Prospective Cohorts of Adults
Source: JMIR Public Health Surveill. 2024 Oct 11;10:e55170. doi: 10.2196/55170 (PMC11512133; doi:10.2196/55170)
Supplement: Multimedia Appendix 9 [file publichealth_v10i1e55170_app9.docx]

| **Table S7**. Demographic characteristics of the Beiwe Smartphone Substudy of Nurses' Health Study 3 (NHS3) and Growing Up Today Study (GUTS) with the breakdown of phone operating system (OS). |
| --- |

|  |  | **By Phone OS** | | |
| --- | --- | --- | --- | --- |
| **Variable** | **Overall, N = 2,394** | **Android, N = 645** | **iOS, N = 1,743** | **Both^a^, N = 6** |
| **Cohort, n (%)** |  |  |  |  |
| Participants from GUTS | 691 (29%) | 217 (34%) | 472 (27%) | 2 (33%) |
| Participants from NHS3 | 1,703 (71%) | 428 (66%) | 1,271 (73%) | 4 (67%) |
| **Age (years), Mean (SD)** |  |  |  |  |
| Mean (SD) | 41.8 (8.1) | 41.0 (8.0) | 42.1 (8.1) | 40.4 (9.1) |
| Median (IQR) | 40.4 (35.4, 49.0) | 39.1 (34.8, 48.1) | 41.3 (35.7, 49.3) | 39.2 (32.9, 47.7) |
| **Sex, n (%)** |  |  |  |  |
| Male | 147 (6.1%) | 51 (7.9%) | 94 (5.4%) | 2 (33%) |
| Female | 2,247 (94%) | 594 (92%) | 1,649 (95%) | 4 (67%) |
| **Race, n (%)** |  |  |  |  |
| White | 2,243 (94%) | 603 (93%) | 1,636 (94%) | 4 (67%) |
| Black or African American | 51 (2.1%) | 16 (2.5%) | 35 (2.0%) | 0 (0%) |
| American Indian or Alaska Native | 22 (0.9%) | 3 (0.5%) | 19 (1.1%) | 0 (0%) |
| Asian | 45 (1.9%) | 10 (1.6%) | 34 (2.0%) | 1 (17%) |
| Native Hawaiian or Other Pacific Islander | 7 (0.3%) | 4 (0.6%) | 3 (0.2%) | 0 (0%) |
| **Ethnicity, n (%)** |  |  |  |  |
| Hispanic or Latino | 80 (3.3%) | 17 (2.6%) | 63 (3.6%) | 0 (0%) |
| Not Hispanic or Latino | 2,307 (96%) | 625 (97%) | 1,676 (96%) | 6 (100%) |
| **Married, n (%)** | 1,437 (60%) | 384 (60%) | 1,051 (61%) | 2 (40%) |
| **Smoking Status, n (%)** |  |  |  |  |
| Never | 1,817 (76%) | 470 (73%) | 1,341 (77%) | 6 (100%) |
| Current | 103 (4.3%) | 40 (6.2%) | 63 (3.6%) | 0 (0%) |
| Former | 468 (20%) | 132 (20%) | 336 (19%) | 0 (0%) |
| **Body Mass Index (kg/m^2^), Mean (SD)** |  |  |  |  |
| Mean (SD) | 27.4 (6.7) | 28.4 (7.3) | 27.0 (6.4) | 22.8 (4.2) |
| Median (IQR) | 25.8 (22.5, 30.6) | 26.5 (23.2, 32.3) | 25.6 (22.3, 30.0) | 21.3 (20.3, 23.2) |
| **Annual household income, n (%)** |  |  |  |  |
| Less than $30,000 | 88 (3.7%) | 30 (4.7%) | 57 (3.3%) | 1 (17%) |
| $30,000 to $50,000 | 118 (4.9%) | 49 (7.6%) | 69 (4.0%) | 0 (0%) |
| $50,000 to $70,000 | 247 (10%) | 80 (12%) | 166 (9.5%) | 1 (17%) |
| $70,000 to $90,000 | 309 (13%) | 109 (17%) | 200 (11%) | 0 (0%) |
| $90,000 to $200,000 | 1,119 (47%) | 272 (42%) | 843 (48%) | 4 (67%) |
| More than $200,000 | 284 (12%) | 50 (7.8%) | 234 (13%) | 0 (0%) |
| Missing/Not Provided | 229 (9.6%) | 55 (8.5%) | 174 (10.0%) | 0 (0%) |

^a^ Some participants switched smartphones during the one-year data collection period, which resulted in a different OS.
